# Maternal prebiotic supplementation impacts colitis development in offspring mice

**DOI:** 10.3389/fnut.2022.988529

**Published:** 2023-01-05

**Authors:** Amélie Lê, Amandine Selle, Philippe Aubert, Tony Durand, Carole Brosseau, Philippe Bordron, Erwan Delage, Samuel Chaffron, Camille Petitfils, Nicolas Cenac, Michel Neunlist, Marie Bodinier, Malvyne Rolli-Derkinderen

**Affiliations:** ^1^The Enteric Nervous System in Gut and Brain Disorders, Institut des Maladies de l’Appareil Digestif, Institut National Pour la Santé et la Recherche Médicale, Nantes Université, Nantes, France; ^2^Unité de Recherche 1268 Biopolymères Interactions Assemblages, Institut National de Recherche pour l’Agriculture, l’Alimentation et l’Environnement, Nantes, France; ^3^UMR 6004, LS2N, Nantes Université, Ecole Centrale Nantes, CNRS, Nantes, France; ^4^UMR 1220, Institut de Recherche en Santé Digestive, Toulouse, France

**Keywords:** prebiotics, IBD, microbiota, colitis, DOHaD, offspring, gut

## Abstract

**Background and aims:**

Maternal diet plays a key role in preventing or contributing to the development of chronic diseases, such as obesity, allergy, and brain disorders. Supplementation of maternal diet with prebiotics has been shown to reduce the risk of food allergies and affect the intestinal permeability in offspring later in life. However, its role in modulating the development of other intestinal disorders, such as colitis, remains unknown. Therefore, we investigated the effects of prebiotic supplementation in pregnant mice on the occurrence of colitis in their offspring.

**Materials and methods:**

Offspring from mothers, who were administered prebiotic galacto-oligosaccharides and inulin during gestation or fed a control diet, were subjected to three cycles of dextran sulphate sodium (DSS) treatment to induce chronic colitis, and their intestinal function and disease activity were evaluated. Colonic remodelling, gut microbiota composition, and lipidomic and transcriptomic profiles were also assessed.

**Results:**

DSS-treated offspring from prebiotic-fed mothers presented a higher disease score, increased weight loss, and increased faecal humidity than those from standard diet-fed mothers. DSS-treated offspring from prebiotic-fed mothers also showed increased number of colonic mucosal lymphocytes and macrophages than the control group, associated with the increased colonic concentrations of resolvin D5, protectin DX, and 14-hydroxydocosahexaenoic acid, and modulation of colonic gene expression. In addition, maternal prebiotic supplementation induced an overabundance of eight bacterial families and a decrease in the butyrate caecal concentration in DSS-treated offspring.

**Conclusion:**

Maternal prebiotic exposure modified the microbiota composition and function, lipid content, and transcriptome of the colon of the offspring. These modifications did not protect against colitis, but rather sensitised the mice to colitis development.

## Graphical Abstract

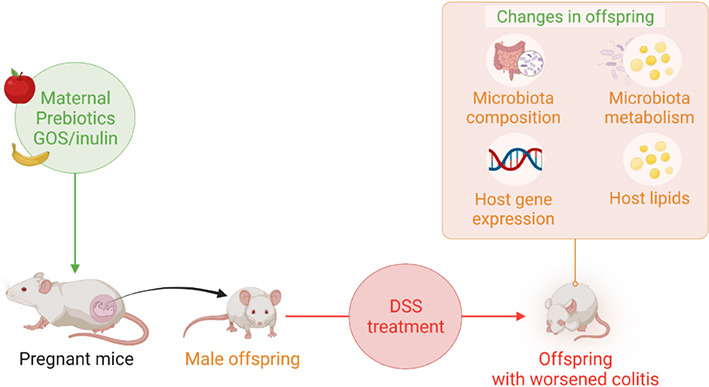

## Introduction

The developmental origins of health and disease paradigm suggests that maternal exposure to environmental factors can have a long-term reprogramming effects on children, which can negatively or positively influence the development of diseases later in life (1). However, limited data are available on whether beneficial environmental factors, such as breastfeeding or maternal diet, can prevent the development of colitis later in life (2–4). Prenatal factors can contribute to *in utero* foetal programming by influencing gene expression and impacting the development of the organs (1, 5), such as the immune system or gut microbiota (6), of the offspring. Maternal diet is an important environmental factor in the development or prevention of diseases in children. For example, a maternal high-fat diet increases the risk of developing cardiovascular diseases in offspring (1). In contrast, maternal intake of vitamin D seems to be associated with protection against allergies in children (7). Therefore, prenatal environmental factors, such as maternal diet, constitute an interesting field of study to better understand the pathogenesis of chronic diseases and potentially contribute to the development of preventive strategies.

During pregnancy, the diet can be modulated by different substrates, such as prebiotics, to influence the health of the mother and foetus. Prebiotics are specific substrates that modulate the gut microbiota and biological systems of the host (8). Several studies have investigated the effects of prebiotics as a maternal dietary intervention on the development of chronic diseases in progeny. For instance, maternal supplementation of mice with prebiotic galacto-oligosaccharides (GOSs) and inulin exerted a protective effect against the development of food allergies in the offspring, with modulation of the gut microbiota, intestinal permeability, and immune responses of the offspring (9, 10). To date, the ability of maternal prebiotic supplementation to modulate the offspring susceptibility to other pathological processes, such as intestinal inflammation, remains unexplored.

Inflammatory bowel disease (IBD), including ulcerative colitis (UC) and Crohn’s disease (CD), is a global health issue that affected approximately 1.5 million people in the USA in Zhu et al. (11), Gajendran et al. (12). IBD affects young adults and children, resulting in an altered immune and enteric nervous system, dysfunction of the gut epithelial barrier, and dysbiosis (13–16). IBD pathogenesis is sustained by: (1) an immune disorder that involves both innate and adaptive cells (17, 18), (2) increased permeability of the gut and defects in epithelial repair (19–23), and (3) a decrease in the gut microbiota diversity and abundance of beneficial strains, such as *Faecalibacterium prausnitzii*, resulting in an alteration of biological functions (24, 25). In addition, the lipid profiles of patients with CD and UC differ from those of the controls, underlining their potential role in IBD pathogenesis (26). While the origin of these dysfunctions could involve genetic and environmental factors, the period of life during which these environmental factors can have a such determining impact remains unknown. Increasing evidence suggests that IBD has, at least partially, a developmental origin (27). This is supported by studies showing that several perinatal environmental factors, such as maternal exposure to antibiotics, tobacco smoke, and early life otitis media, are associated with a high risk of IBD for the offspring later in life (3).

Thus, the aim of our study was to uncover the consequences of maternal supplementation with the prebiotics, GOSs and inulin, during gestation on the development of colitis in offspring mice. We have previously shown that maternal supplementation with GOSs and inulin induces a modification in the gut microbiota of mouse mothers (6). This modification was associated with an increase in the production of short-chain fatty acids (SCFAs), especially acetate and propionate (6), in stools, which was maintained until the mid-lactation period (10). This maternal prebiotic supplementation also changed the gut microbiota composition and gut metabolite production of the 6-weeks-old female offspring, inducing a tolerogenic environment mitigating food allergy (10). In the present study, we investigated whether imprinting could delay colitis induced by dextran sulphate sodium (DSS) in 8-weeks-old offspring mice. In addition to the assessment of intestinal function and disease activity, we investigated the mechanisms underlying the effects of maternal prebiotic supplementation on colitis susceptibility by studying the gut microbiota composition, colonic gene expression, and bioactive lipid profile. Among the bioactive lipids, we analysed oxylipins that are involved in inflammatory processes (28), and regulate different gut properties that are defective or overactivated in IBD (16, 23, 26, 29). The results of our study broaden the existing knowledge on the determinant role of maternal diet, especially in the development of IBD in offspring.

## Materials and methods

### Animal studies


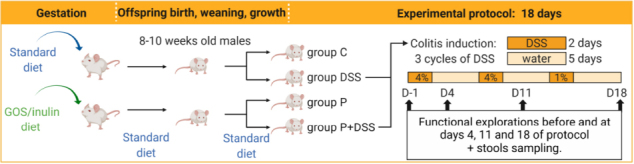


Eight-week-old BALB/cJRj mice from Janvier Labs (Le Genest-Saint-Isle, France) were housed in a ventilated cage system under a 12-h light-dark cycle. The study protocol was approved by the Ethics Committee on Animal Experimentation of the Pays de la Loire region (accession number: 16925). Mice were fed either a standard diet (abbreviated C) or a diet (abbreviated P) supplemented with 4% GOSs (DOMO VIVINAL GOS powder; Friesland-Campina, The Netherlands) and inulin (Orafti HP; BENEO-Orafti, Belgium) in a 9:1 ratio, with the mass percentage of other sugars lowered to reach the same total sugar mass percentage as the standard diet (Safe, France). The formulation of the supplemented diet was validated in previous studies by a research group (30). Water and food were provided *ad libitum*. In total, the prebiotic exposure lasted 5 weeks (including 1 week of acclimation, 1 week of mating, and three weeks of gestation). For all experiments, after delivery, the mothers and pups were fed a standard diet. Mice were monitored daily and euthanised if the limit points (including prostration and >20% weight loss) were reached.

For the mouse model of colitis, 8–10-weeks-old male offspring from each group of mothers (exposed or not exposed to prebiotics) were subjected to three cycles of DSS (MW = 36,000–50,000 Da; MP Biomedicals) treatment. One cycle consisted of 2 days of providing 4 or 1% at the last cycle, of DSS in drinking water (renewed each day) followed by 5 days of providing only water. This chronic DSS protocol was adapted from the work of Creyns et al. to retain the chronicity of three administrations within a shorter induction period (31). In the end, four groups of mice were distinguished: the mice of mothers who followed a standard diet either exposed to DSS (DSS) or left untouched (C), and the mice of mothers supplemented with prebiotics either exposed to DSS (P + DSS) or left untouched (P). Animals were weighed daily during the protocol, D1 corresponding to 1 day after starting the first DSS administration. *In vivo* functional explorations were conducted before (D-1) and during the protocol on days 4, 11, and 18 (D4, D11, and D18, respectively). Mice showing two significant clinical signs (10% weight loss, cold to touch, prostration, closed eyes, and shaggy hair) or one severe clinical sign (20% weight loss) were removed from the study and euthanised.

Survival curves were generated, and at the end of the protocol on D18, mice were killed via cervical dislocation. The colon length of each mouse was measured from the end of the caecum to the anus. Intestinal fragments (caecum, proximal, and distal colon), associated luminal contents, and faeces were collected and snap-frozen for omics, lipocalin-2 (Lcn-2), or SCFA analyses. Intestinal fragments were also opened along the mesenteric border for *ex vivo* permeability assessment or fixed for at least 1 h in paraformaldehyde solution [4% in phosphate-buffered saline (PBS)] for immunohistochemistry and histopathology. The project included three independent protocol repeats, with 8–12 mice per group for each protocol. The number of analysed animals was specified for each analysis.

### *In vivo* intestinal functional exploration

Intestinal functional exploration consisted of the macroscopic characterisation of colitis development assessed by the evaluation of intestinal permeability, disease activity index (DAI), faecal pellet output (FPO), total transit time (TTT), and stool humidity. Mice received (5 μl/g of mouse) a solution comprising 60 mg/ml carmine red (cochineal carmine, Prolabo #22259), 10 mg/ml fluorescein-5,6-sulfonic acid (FSA; Thermo Fisher Scientific, Waltham, MA, USA), and 10 mg/ml horseradish peroxidase (HRP; Sigma-Aldrich) resuspended in 0.5% carboxymethylcellulose in PBS (Sigma-Aldrich) via gavage, and then placed in individual cages.

#### Intestinal permeability

Four hours after gavage, blood was sampled from the tail vein in vials containing PBS-EDTA to isolate the plasma via centrifugation at 3,200 rpm for 5 min at 20°C. Paracellular permeability was evaluated *via* titration of FSA fluorescence intensity in plasma and measured using an automatic microplate reader (Varioskan; Thermo Fisher Scientific, Waltham, MA, USA). These plasma samples were kept frozen and later used to determine the transcellular permeability to HRP using an enzymatic activity assay with 3,3′,5,5′-tetramethylbenzidine reagent (BD Biosciences).

#### Disease activity index

The disease activity index (DAI) was calculated based on the stool consistency observed immediately after gavage (0 = hard faeces, 1 = soft faeces, 2 = liquid faeces), blood in faeces (0 = no blood, 1 = blood), and weight loss (0 = no loss, 1 = <5% loss, 2 = loss of 5–10%, 3 = loss of 10% and more). The DAI ranged from 0 to 6.

#### Faecal pellet output, total transit time, faeces humidity

Faecal pellet output (FPO) represented the number of faeces emitted by each mouse over 2 h. Total transit time (TTT) corresponded to the interval between gavage and the first observation of red carmine faeces in each mouse. For humidity assessment, the difference in weight between dried and fresh FPO faeces was calculated and divided by the weight of fresh FPO faeces (faeces humidity).

### Lipocalin-2 enzyme-linked immunosorbent assay faecal quantification

The faeces of each mouse was resuspended in a solution of PBS-Tween 20 0.1% protease inhibitor cocktail (1/2 tablet for 25 ml solution, cOmplete; Roche) to reach 100 mg faeces/ml. Lcn-2 in the faecal solution was measured using an enzyme-linked immunosorbent assay (ELISA) kit (Mouse Lcn-2/NGAL DuoSet ELISA; Bio-Techne), according to the manufacturer’s protocol.

### *Ex vivo* intestinal permeability assessment with Ussing chambers

The *ex vivo* paracellular and transcellular permeabilities of the collected intestinal segments were measured as previously described (26). Mice intestinal segments were mounted in Ussing chambers (0.03 cm^2^ exposed surface area; Physiologic Instruments, San Diego, CA, USA). Each Ussing chamber was filled with 2 ml of Ham’s/F12 medium (Invitrogen) maintained at 37°C and continuously gassed with 95% O_2_ and 5% CO_2_. Following an equilibration time of 30 min, 200 μl of apical medium was replaced by 200 μl of Ham’s/F12 medium containing FSA (1 mg/ml) and HRP (1 mg/ml). The fluorescence level of basolateral aliquots (150 μl) taken every 30 min for a period of 150 min was measured to evaluate paracellular permeability, using the automatic fluorescence microplate reader (Varioskan; Thermo Fisher Scientific, Waltham, MA, USA) at 485 nm excitation and 520 nm emission wavelengths. From these basolateral aliquots, 30 μl were then taken to measure the transcellular permeability to HRP using the enzyme activity assay with 3,3′,5,5′-tetramethylbenzidine reagent (BD Bioscience) (32).

### Immunohistochemistry and histopathology

Four percent paraformaldehyde-fixed distal colon tissues were embedded in paraffin and sectioned into 5-μm thick slices with a microtome (HM 355S; Thermo Fisher Scientific, Waltham, MA, USA) either using the Therassay platform (Nantes, France) or MicroPICell platform (Nantes, France).

#### Histological score: Micro disease activity index

Slides were stained with haematoxylin–phloxine–saffron or alcian blue, using the MicroPICell platform or the Inserm UMR1238 Phy-OS laboratory (Nantes, France), to visualise the tissue morphology and goblet cells. Images were taken using a slide scanner (Nanozoomer; Hamamatsu). As previously described, distal colonic tissue damage was evaluated in a blinded manner through the calculation of a micro disease activity index (micro DAI) which reflects the destruction of mucosal architecture, the cellular infiltration, muscle thickening and goblet cells depletion (26). The degree of destruction of normal mucosal architecture was scored as 0–3 (0 = no destruction, 1 = 1/3 basal destruction, 2 = 2/3 basal destruction, 3 = loss of crypt and epithelium). The extent of cellular infiltration was scored as 0–3 when the infiltration was, respectively, normal, around the crypt basis, reaching the muscularis mucosae or reaching the submucosa. The degree of muscle thickening was also ranging from 0 to 3, when the thickening was none, mild, moderate, or massive, respectively. The loss of goblet cell depletion was scored as 0 (normal presence) or 1 (massive depletion). A multiplication factor of 1–4 was applied for each measured criteria, depending on the extent of affected area (25, 50, 75, or 100%) of the considered sample.

#### Immunostaining

Paraffin was removed from the slides using two xylene baths (10 min) followed by four ethanol baths: 100% for 5 min, 95% for 4 min, 70% for 3 min, and 50% for 3 min. After washing the slides in PBS, antigen retrieval was performed via incubation at 95°C for 20 min in sodium citrate solution (10 mM sodium citrate tribase-0.05% Tween 20 in distilled water, pH = 6). The slides were incubated in NH_4_Cl solution (100 mM) for 30 min, followed by permeabilization in a PBS-NaN_3_-1% saponine-0.5% Triton X-100 solution for 30 min to 1 h. A blocking period of 1 h for overnight incubation was performed in PBS-NaN_3_-1% saponine-0.5% Triton X-100-10% horse serum. The primary antibodies for CD3 (1:1,000, rabbit polyclonal antibody; Abcam) and F4/80 (1:500, rat monoclonal antibody; Cell Signalling Technology) were used overnight at 4°C. After three 15-min washes in PBS, secondary antibodies (1:500, donkey Cy3-labelled anti-rabbit and anti-mouse antibodies) were incubated for 2 h at room temperature. Slides were washed thrice for 15 min in PBS and once for 5 min with 4, 6-diamino-2-phenylindole (1:1,000 in PBS). Coverslips were mounted using ProLong Gold Antifade Mountant (Thermo Fisher). Images were acquired with an Axio Zoom.V16 microscope (Zeiss) and analysed using the ImageJ/Fiji software (National Institute of Health, Bethesda, Maryland, USA).

### Transcriptomic analyses

Distal colon samples were lysed in RA1 buffer (Macherey-Nagel) before total RNA extraction using the NucleoSpin RNA kit, according to the manufacturer’s instructions (Macherey-Nagel). Purified mRNA was used for sequencing with the 3′ seq-RNA profiling (3′ SRP) protocol performed using the GenoBiRD platform (Nantes, France) (33). Sequencing data were deposited with the ArrayExpress accession number E-MTAB-12176. During primary analysis, reads were stripped of any poly A tails using cutadapt (version 1.18) and then aligned to RefSeq mouse mRNA sequences (mm10) using BWA (version 0.7.17, with the non-default parameter “–l 24”) (34, 35). Reads mapped to several positions in the genome were filtered out from the analysis. Secondary analysis was performed using samples that had more than 4,50,000 reads assigned and more than 5,000 genes detected (*i.e.*, the number of genes with at least one count). From the initial 61 samples, 57 samples were kept after removing one sample from the DSS group and three samples from the P + DSS group. We then removed genes with no counts from at least 13 samples. Principal component analysis (PCA) was performed using the 3′ SRP pipeline, which relies on the *prcomp* function in R. Differential analysis was conducted using DESeq2 (version 1.26.0) to normalise gene counts and study the differentially expressed genes (DEGs) (36). DEGs between the two conditions were genes with an adjusted *p*-value < 0.05 and an absolute value of gene expression fold-change >1.5. The following comparisons were performed: P + DSS *vs.* DSS, P + DSS *vs.* P, DSS *vs.* C, and P *vs.* C. An indirect analysis was also conducted for P + DSS *vs.* DSS by looking at specific and common DEGs obtained from the comparisons P + DSS *vs.* P and DSS *vs.* C, *that is* DEGs that only existed in P + DSS *vs.* P or DSS *vs.* C, and DEGs that existed in both P + DSS *vs.* P and DSS *vs.* C groups. Finally, over-representation enrichment analysis was conducted on the list of DEGs using clusterProfiler (version 3.14) with Gene Ontology (GO) and Kyoto Encyclopedia of Genes and Genomes (KEGG) knowledge bases (37). For each comparison, the universe used with the enrichGO or enrichKEGG functions is the set of genes (DEGs and not DEGs) present in the DESeq2 results table. The biological enrichments obtained from each comparison were compared using the *emaplot* function from clusterProfiler. In this case, the universe was the union of the universes used in each comparison.

### Polyunsaturated fatty acid metabolite profiling

Polyunsaturated fatty acid (PUFA) metabolites were quantified in mouse colons via MS after lipid extraction, as previously described (38). After the addition of 500 μl of PBS and 5 μl of deuterated internal standard (IS) mixture (5-HETEd8, LxA4d4, and LtB4d4), the colons were crushed by lysing MatrixA tubes in Precellys (Bertin Technologies). Following two crush cycles (6.5 ms to 1.30 s), 10 μl of suspension was withdrawn for protein quantification and 0.3 ml of cold methanol (MeOH) was added. The samples were centrifuged at 1,016 × *g* for 15 min (4°C) and the resulting supernatants were subjected to solid-phase lipid extraction using an HLB plate (OASIS HLB 30 mg, 96-well plate; Waters, Saint-Quentin-en-Yvelines, France). Briefly, the plates were conditioned with 500 μl MeOH and 500 μl H2O/MeOH (90:10, v/v). The samples were loaded at a flow rate of approximately 1 drop per 2 s, and after complete loading, the columns were washed with 500 μl H2O/MeOH (90:10, v/v). The phases were thereafter dried under aspiration, and the lipids were eluted with 750 μl of MeOH. The solvent was evaporated under N_2_, and the samples were resuspended in 140 μl of MeOH and transferred into a vial (Macherey-Nagel, Hoerdt, France). Finally, 140 μl of methanol was evaporated and each sample was resuspended in 10 μl of methanol for liquid chromatography-tandem mass spectrometry (LC-MS/MS) analysis. The 53 lipids quantified in mouse colons are listed in Supplementary Figure 1. To simultaneously separate these 53 lipids of interest and three deuterated ISs (5-HETEd8, LxA4d4, and LtB4d4), LC-MS/MS analysis was performed on an ultrahigh-performance liquid chromatography system (UHPLC; Agilent LC1290 Infinity) coupled to an Agilent 6460 triple quadrupole MS (Agilent Technologies) equipped with electrospray ionisation operating in negative mode. Reverse-phase UHPLC was performed using a Zorbax SB-C18 column (Agilent Technologies) with gradient elution. The mobile phases consisted of water, acetonitrile (ACN), and formic acid (FA) [75:25:0.1 (v/v/v)] (solution A) and ACN and FA [100:0.1 (v/v)] (solution B). The linear gradient was as follows: 0% solution B at 0 min, 85% solution B at 8.5 min, 100% solution B at 9.5 min, 100% solution B at 10.5 min, and 0% solution B at 12 min. The flow rate was 0.4 ml/min. The autosampler was set at 5°C and the injection volume was 5 μl. Data were acquired in multiple reaction monitoring mode under optimised conditions. Peak detection, integration, and quantitative analysis were performed using MassHunter Quantitative analysis software (Agilent Technologies). Blank samples were evaluated and their injection showed no interference (no peak detected) during the analysis. TxB_2_, RVD2, LxA4, and 7Mar1 were not detected in the samples. MetaboAnalyst web interface^1^ was used for data analysis. The data were first scaled using an auto-scaling method to obtain mean-centred values divided by the standard deviation of each variable. A heatmap was generated with hierarchical clustering of group averages and metabolites, using the average clustering algorithm and the Euclidean distance measure of the scaled data.

### Gut microbiota analysis

#### Composition

##### Sample preparation and sequencing

The luminal contents of the distal colon and caecum of each mouse were collected and snap-frozen.

##### Metabarcoding analysis

DNA extraction of the luminal contents of the distal colon and sequencing on an Illumina MiSeq platform were performed by Biofortis (Nantes, France). Raw sequencing data were obtained from a single run as 250 bp paired-end reads targeting the V3–V4 region (primers: Bakt_341F 5′-CCTACGGGNGGCWGCAG-3′ and Bakt_805R 5′-GACTACHVGGGTATCTAATCC-3′) of the *16S rDNA* gene. Sequencing data were deposited under the ENA accession number, PRJEB55683. Reads were processed with microSysMics,^2^ a workflow built around the QIIME2 toolbox (version 2020.6), to streamline the metabarcoding analysis (39, 40). Polymerase chain reaction primers and the remaining Illumina adapters were removed using Cutadapt. Amplicon sequence variant (ASV) inference, and count estimation was performed with DADA2 using a trimming length of 220 and the default parameters (41). We used a naive Bayes classifier pre-trained on the SILVA 99% reference database (Release 138) to taxonomically annotate ASVs. Alpha- and beta-diversity metrics were calculated using a rarefied ASV table (sampling depth: 52,305 reads). Shannon index and Bray–Curtis dissimilarity boxplots were produced using R package ggstatsplot (version 0.7.0) with Kruskal–Wallis, followed by pairwise Dunn used as statistical tests (42). Principal coordinate analysis (PCoA) was also performed for Bray–Curtis dissimilarity. Permutational multivariate analyses of variance (PERMANOVA) analysis was conducted with the R package vegan (version 2.5-7) using the adonis function set to 9,999 permutations (43). Differential abundance analysis was performed using DESeq2 (version 1.30.1) (36). A pre-filtering step was applied to removed ASVs with a low prevalence (found in less than five samples). Normalisation was done in “poscounts” mode, which accounts for the inherent sparsity of sequencing data. The Benjamini–Hochberg procedure with a false-discovery rate of 0.05 was applied to account for multiple testing, resulting in adjusted *p*-values.

#### Short-chain fatty acids measurement

Measurement of the levels of the short-chain fatty acids (SCFAs) acetate, butyrate, isobutyrate, valerate, and propionate, was performed in the caecal contents of mice at the Mass Spectrometry Core Facility of CRNH-O (Nantes, France) (44). Briefly and as exactly previously described, stock solutions of SCFA metabolites (Sigma-Aldrich, Saint-Quentin-Fallavier, France) were diluted to get ten calibration solutions (44). A working solution of internal standards was prepared in 0.15 M sodium hydroxide to get the following final concentrations: 75 mmol/L of D3-acetate, 3.8 mmol/L of D5-propionate, 2.5 mmol/L of 13C-butyrate, and 0.5 mmol/L of D9-valerate (Sigma-Aldrich). Caecal content samples (100 μl) were dissolved in 200 μl of sodium hydroxide solution at 0.15 M (Sigma-Aldrich) (44). Twenty microliters of the internal standard solution were added to samples and calibration solutions (44). After addition of the standards, each sample was acidified with 5 μl of hydroxide chloride 37% (Sigma-Aldrich) and then extracted with 1.7 ml of diethyl ether (Biosolve, Dieuze, France) (44). Samples were stirred gently for 1 h and then centrifuged 2 min (5,000 rpm, 4°C) (44). The organic layers were transferred into 1.5 ml glass vials and SCFAs were derivatized with 20 μl of tert-butyldimethylsilyl imidazole (Sigma-Aldrich). Samples were incubated for 30 min at 60°C before analysis. Samples were finally analyzed by gas chromatography–mass spectrometry (model 7890A-5975C; Agilent Technologies, Montpellier, France) using a 30 m × 0.25 mm × 0.25 μm capillary column (HP1-MS; Agilent Technologies) (44). The temperature program started at 50°C for 1 min, ramped to 90°C at 5°C/min and then up to 300°C at 70°C/min. Selected ion monitoring mode was used to measure SCFA concentrations with ions at 117 m/z (acetate), 120 m/z (D3-acetate), 131 m/z (propionate), 136 m/z (D5-propionate), 145 m/z (butyrate and isobutyrate), 146 m/z (13C-butyrate), 159 m/z (valerate), and 168 m/z (D9-valerate) (44).

### Statistical analysis

For omic analyses, immunohistochemistry, and SCFA quantification, 57–78 samples, according to the analysis, were picked randomly using the Excel functions, RAND and RANK. Statistical analyses and associated plots were generated using GraphPad Prism software (La Jolla, CA, USA). Additional statistical tests were performed on the omic data. Data are expressed as the mean standard deviation, and were mainly compared by two-way analysis of variance corrected by a Bonferroni multiple-comparisons test or log-rank test for survival analysis. Normalisation by control group was performed according to each experimental series. No significant results are shown in the graphics. Values were considered significant if the adjusted *p*-value < 0.05.

## Results

### Maternal prebiotic supplementation impacts colitis in the offspring

**FIGURE 1:**
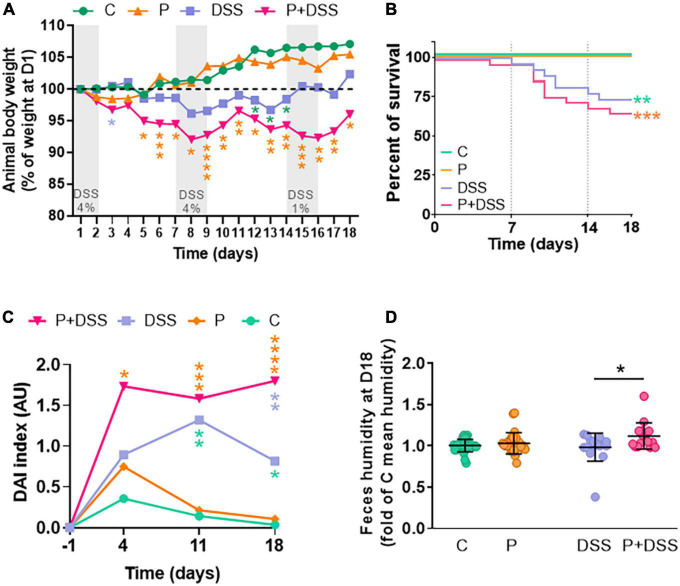
Prebiotic administration to mothers sensitises their offspring to colitis. Impact of prebiotic administration to mothers was measured in offspring during and at the end of a protocol of colitis induction by dextran sulphate sodium (DSS) in all groups of mice. Pups of mothers fed with a standard diet were administered DSS (in blue) or left untouched (in green). The same procedure was followed for pups of mothers fed a diet enriched with prebiotics (P + DSS in pink, P in orange). **(A)** Weight curves of the animals, where the weight was normalised as per the weight on the first day of DSS treatment (D1) for each mouse. *N* = 28–30 mice/group. **(B)** Survival curves of mice. *N* = 26–30 mice/group. **(C)** DAI was measured before (D-1) and on D4, D11, D18 of the protocol. *N* = 29–30 mice/group. **(D)** Faeces humidity for each mouse. *N* = 16–28 mice/group. Data represent the mean and standard deviation of the mean. Log-rank test for the survival rate or two-way analysis of variance (ANOVA), followed by Bonferroni’s *post hoc* comparisons tests: *p < 0.05, **p < 0.01, ***p < 0.001, ****p < 0.0001, blue asterisk for P + DSS *vs.* DSS, orange for P + DSS *vs.* P, and green for DSS *vs.* C groups.

To assess the consequences of maternal prebiotic supplementation with GOSs and inulin on colitis development in the offspring, chronic colitis was induced in 8–10-weeks-old male offspring from both standard diet-fed mothers (C) and prebiotic-supplemented mothers during gestation (P) by DSS administration. Weight was monitored daily, and a similar increase in weight was observed between the C and P groups (Figure 1A). The weight of the DSS group was significantly lower at the end of the second DSS cycle than that of the C group (Figure 1A). The weight of the animals from the P + DSS group was significantly lower than that of animals from the P group from D5–D18 of the protocol. The weight of the animals from the P + DSS group was significantly lower than that of the DSS group on D3 (Figure 1A). Along with the weight, the survival of the animals was evaluated. The survival curves of the P + DSS and DSS groups were similar (Figure 1B). Increased DAI was observed at the end of the first, second, and third DSS cycles (D4, D8, and D11) for the P + DSS *vs.* P group, and only at the end of the second and third DSS cycles (D8, D11) for the DSS *vs.* C group (Figure 1C). The difference in DAI between the P + DSS and DSS group only reached significance on D18 (1.8 *vs.* 0.8 AU, Figure 1C and Supplementary Figure 2A). No difference of DAI was observed for the P *vs*. C group during the protocol (Figure 1C). Regarding physiological parameters, DSS treatment or maternal prebiotic supplementation alone did not affect, for example, faeces humidity (Figure 1D). We observed however, on D18, a significant increase of mean faecal humidity for the P + DSS *vs.* DSS groups (1.12 *vs.* 0.984-fold, Figure 1D). Concerning other parameters on D18, TTT was significantly increased and FPO was significantly reduced in DSS-treated mice compared to their respective controls (Supplementary Figures 2B,C). No significant differences were observed between the DSS-treated groups (Supplementary Figures 2B,C). However, it should be noted that TTT on D4 was significantly higher in the P + DSS group than in the DSS group, again showing the increased sensitivity of the offspring from prebiotic-supplemented mothers (Supplementary Figure 2D). Finally, maternal prebiotic supplementation did not change the *in vivo* intestinal paracellular permeability compared to the C group on D18, but it protected against the DSS-induced increase in paracellular permeability (increase in the DSS *vs*. C groups, no difference for the P + DSS *vs*. P groups, Supplementary Figure 2E). Similarly, on D18, the DSS-induced increase in transcellular permeability in the proximal colon was prevented by maternal prebiotic exposure (lower in the P + DSS vs. DSS groups, 0.863 *vs*. 1.08, Supplementary Figure 2F). Altogether, these results suggest that maternal prebiotic supplementation could worsen colitis in the offspring, with a transient decrease in weight loss and increase in TTT, as well as an impact on faecal consistency and an increased DAI at the end of the third DSS cycle.

### Maternal prebiotic supplementation does not change micro-disease activity index but induces immune cell infiltration in the distal colon of dextran sulphate sodium-treated offspring

**FIGURE 2:**
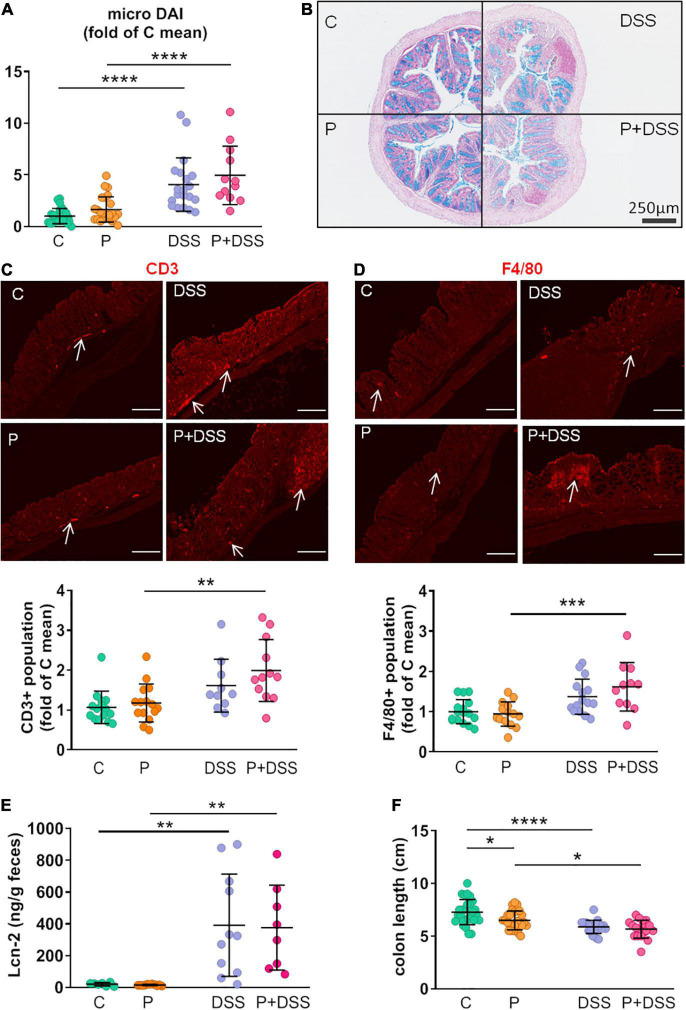
Prebiotic administration to mothers increases colon shortening and DSS-induced immune cell infiltration, but not global inflammation or tissue remodelling, in the offspring. Colon remodelling was evaluated through calculation of the **(A)** histological score (micro–DAI), representing the destruction of mucosal architecture, muscle thickening, loss of goblet cells, and cellular infiltration, after haematoxylin–phloxine–saffron (HPS) and alcian blue staining of distal colon sections (**B** representative pictures of the sections for each group of mice). *N* = 12–25 mice/group. Precise immune cell infiltrations were evaluated via immunostaining of CD3 (T cells; **C**) and of F4/80 (macrophages; **D**) in distal colon sections. *N* = 10–15 and 11–16 mice/group, respectively. Scale bar = 100 μm. Global intestinal inflammation was evaluated in all four groups of mice through the measurement of lipocalin-2 (Lcn-2; **E**) faecal content and colon shortening **(F)**. *N* = 7–11 and 18–27 mice/group, respectively. Data represent the mean and standard deviation of the mean. Two-way ANOVA, followed by Bonferroni’s *post hoc* comparisons tests: *p < 0.05, **p < 0.01, ***p < 0.001, ****p < 0.0001 (Prebiotics or DSS effect).

To further characterise DSS- and/or P-induced intestinal inflammation, we analysed micro-DAI, immune cell infiltration in the distal colon, release of a marker of intestinal injury (faecal concentration of Lcn-2 for neutrophil gelatinase-associated Lcn), and colonic inflammation (colon length). The micro-DAI score increased in both the DSS and P + DSS groups compared to their respective controls, and no significant effect of maternal prebiotic supplementation was observed (Figures 2A,B). The effect of maternal prebiotic supplementation on the immune inflammatory response in control and DSS conditions was then investigated by analysing the innate and adaptive immune cells, such as F4/80 + macrophages and CD3 + T cells in the distal colon. A significant increase in both T cells and macrophages was observed only in the mucosa of the P + DSS *vs.* P groups (2-fold of C mean CD3 + T cell population for the P + DSS group *vs.* 1.2-fold for the P group; 1.6-fold of C mean F4/80 + macrophage population for P + DSS *vs.* 0.9-fold for the P group). The same trend was observed for the DSS *vs.* C groups, although the difference was not statistically significant (Figures 2C,D). No significant differences were observed between the P + DSS *vs.* DSS groups. Furthermore, Lcn-2 faecal concentration was increased in the DSS and P + DSS groups compared to their respective control group (392–377 ng/g faeces in DSS-treated group *vs.* 16–21 ng/g faeces in control group) independent of maternal prebiotic exposure (Figure 2E). DSS administration induced colon shortening in both DSS and P + DSS groups (Figure 2F). Interestingly, maternal prebiotic exposure induced significant colon shortening, even without DSS treatment (6.5 ± 0.9 cm in P group *vs.* 7.3 ± 1.2 cm in C group). These results suggest that basal colonic inflammation is induced by maternal prebiotic supplementation in pups and recruits immune cells only in the P + DSS group.

### Maternal prebiotic supplementation potentially modulates the transcriptomic response to dextran sulphate sodium treatment in distal colon

**FIGURE 3:**
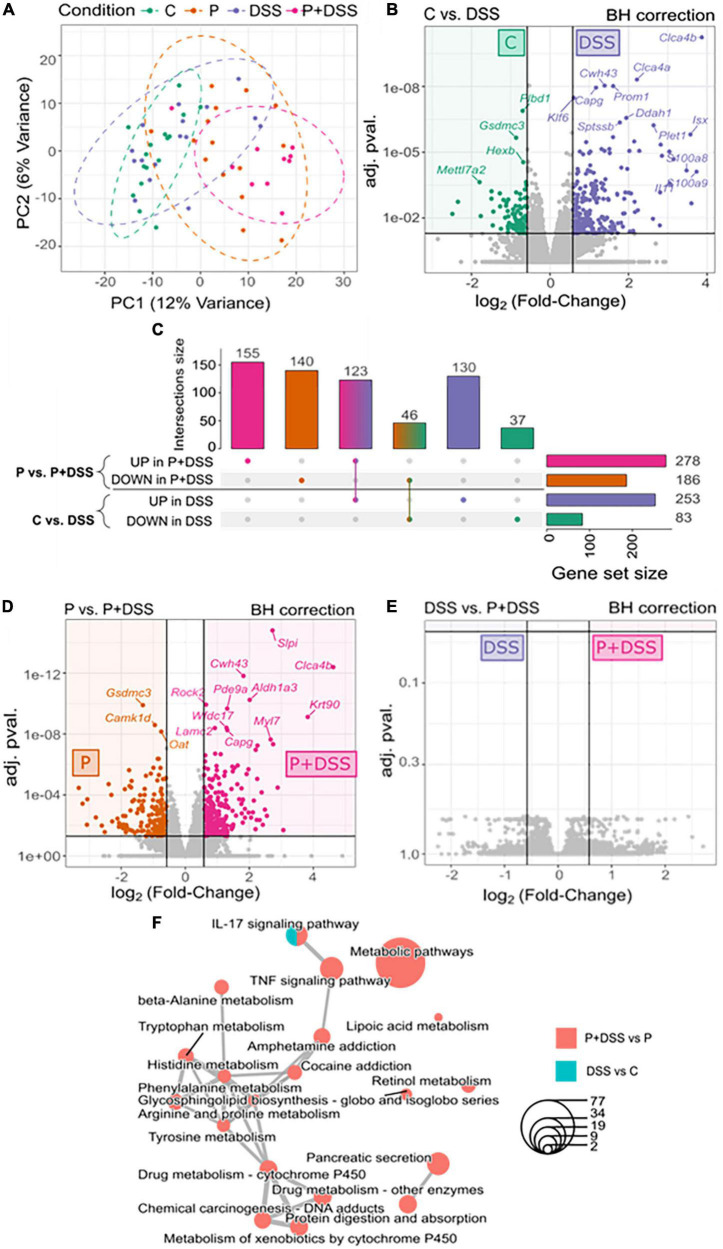
Prebiotic administration to mothers modulates the transcriptomic response to DSS treatment in distal colon: 155 upregulated genes and 140 downregulated genes are specific to the P + DSS group. **(A)** Principal component analysis describes the transcriptomic profile of each mouse in the distal colon. Volcano plots show the differentially expressed genes in the distal colon for comparison **(B)** C *vs.* DSS, **(D)** P *vs.* P + DSS and **(E)** DSS *vs.* P + DSS. Upregulated genes are coloured according to the group of mice (**C**, green; P, orange; DSS, violet; P + DSS, pink). Grey dots show the non-significant differences in gene expression. **(C)** Impact of the maternal prebiotic supplementation on the response to DSS treatment, represented by the comparisons of genes for P *vs.* P + DSS and C *vs.* DSS groups, were compared, and specific and common sets of up- and downregulated genes were identified. These identified genes were comprised in a **(F)** network of enriched pathways from the Kyoto Encyclopedia of Genes and Genomes (KEGG) pathway enrichment analysis. *N* = 13–19 mice/group at the beginning of the analysis.

Next, we aimed to further analyse the impact of maternal prebiotic supplementation on colitis development in the offspring using 3′ SRP transcriptomic profiling approaches. PCA revealed four clusters of transcripts corresponding to the four experimental groups, which encompassed overlapping and distinct parts (Figure 3A). Analysis of differentially expressed genes showed that 253 genes were upregulated and 83 were downregulated in the DSS and C groups, respectively (Figures 3B,C). In the P + DSS group, 278 upregulated genes and 186 downregulated genes were found compared to those in the P group (Figures 3C,D). An over-representation enrichment analysis using the Kyoto Encyclopaedia of Genes and Genomes (KEGG) database showed that in offspring from standard diet-fed mothers, DSS induced changes in genes mainly associated with the regulation of the inflammatory response and lipid metabolism (Supplementary Figure 2A). In offspring from prebiotic-supplemented mothers, DSS treatment induced changes in genes belonging to KEGG modules related to inflammation as well as genes associated with xenobiotics and amino acid metabolism (Supplementary Figure 3B). Interestingly, the number and type of regulated genes following colitis induction differed depending on the maternal diet.

No differentially expressed genes were found between the DSS and P + DSS groups (Figure 3E). Nevertheless, the comparison of both responses to DSS treatment, *that is*, P *vs.* P + DSS and C *vs.* DSS, revealed 155 up- and 140 down-regulated genes identified only in the comparison P *vs.* P + DSS, but not C *vs.* DSS. This result may be linked to the specific effects of maternal prebiotic supplementation (Figure 3C and Supplementary Figures 3C,D). The 155 upregulated genes specific to the P + DSS group included genes associated with immune pathways, such as *Anxa1*, *Cebpb*, *Cxcl1*, *Tnfrsf1b*, *Tnfrsf23* pathways, and epithelial barrier regulation, such as *Clca3b* (Supplementary Figure 3D). The 140 downregulated genes specific to the P + DSS group included genes associated with lipid metabolism, such as *Cyp2s1*, *Hpgds*, and *Ppargc1a*, and epithelial barrier regulation, such as *Aqp11*, *Clcc1*, and *Cldn2.* Genes associated with neurological processes, such as *Maoa* and *Maob*, *Comt*, and *Sema5a*, were also downregulated (Supplementary Figure 3D). KEGG analysis based on this comparison of DSS responses showed enhanced metabolic pathways specific to the P + DSS *vs.* P comparison of transcriptomes and included the TNF signalling pathway (Figure 3F in bold). While the IL-17 signalling pathway was a common module induced in both P + DSS and DSS groups, genes encompassed in the module were different according to the maternal diet, with *Casp8*, *Cebpb*, and *Cxcl1* significantly induced only in the P + DSS group (Supplementary Figures 3A,B). KEGG analysis also highlighted modules related to the metabolism of amino acids, such as tyrosine and tryptophan, among the modules specific to the comparison of P + DSS *vs.* P groups (Figure 3F). Furthermore, maternal prebiotic supplementation induced a change in the transcriptomic profile in basal condition. Thirteen genes were upregulated in the P group compared to the control group and comprised KEGG modules related to sperm development (Supplementary Figures 4A,B). Enrichment analysis based on the Gene Ontology (GO) database also highlighted modules linked to amino acid metabolism (Supplementary Figure 4C).

Taken together, these data indicate that maternal prebiotic supplementation modified the basal transcriptomic profile in offspring, but not the gene expression profile observed after DSS treatment.

### Maternal prebiotic supplementation induces changes in the distal colon lipid profile of the offspring and a switch in lipid production after dextran sulphate sodium treatment

**FIGURE 4:**
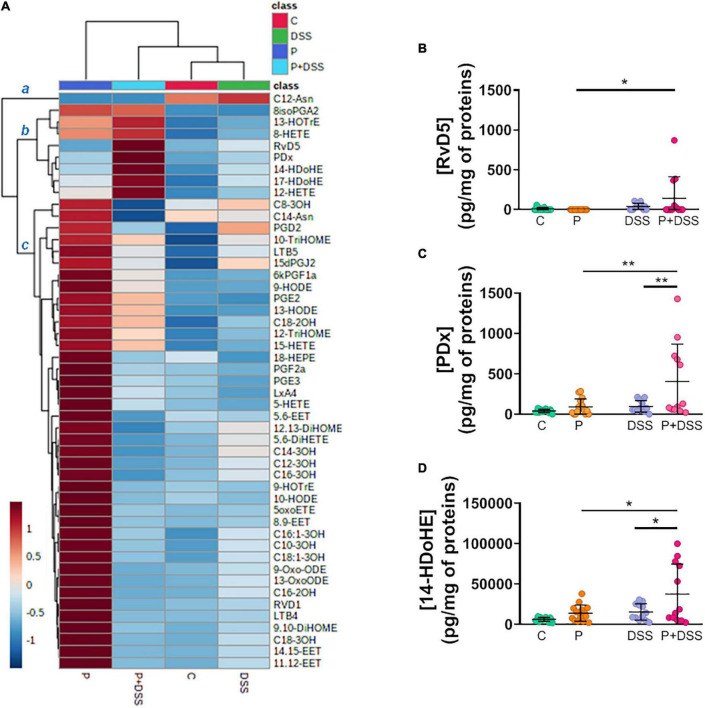
Prebiotic administration to mothers remodels the bioactive lipid profiles and increases the concentrations of resolvin D5, protectin Dx, and 14-hydroxydocosahexaenoic acid after DSS treatment in distal colon. **(A)** Heatmap showing the mean normalised concentrations of 49 lipid mediators in the distal colon of each group of mice identified clusters of metabolites with similar variation in the P + DSS group. The colour gradient indicates the fold-change, with red and blue colours indicating up- and downregulation, respectively. **(B–D)** Analysis of the concentrations of lipid mediators identified in the upregulated *b* cluster: resolvin D5 (RvD5), protectin Dx (PDx), and 14-hydroxydocosahexaenoic acid (14-HDoHE), *via* two-way ANOVA, followed by Bonferroni’s *post hoc* comparisons tests: *p < 0.05, **p < 0.01 (Prebiotics effect). Data represent the mean and standard deviation of the mean of 12–16 mice per group.

We extended our analyses by establishing a bioactive lipid profile of the distal colon by measuring the concentrations of 49 *n* −6/*n* −3 PUFA-derived metabolites and other bacterial long-chain fatty acids. A heatmap enabled the identification of three main clusters of compounds: cluster *a* consisted of C12-Asn produced at low levels in the P and P + DSS groups; cluster *b* consisted of eight compounds produced at high levels in the P + DSS group; and cluster *c* consisted of 40 compounds produced at high levels in the P group (Figure 4A). Indeed, the lipid profile was poorly affected by DSS treatment, whereas maternal prebiotic supplementation completely changed the lipid profile and induced a significant increase in the concentrations of 28 compounds belonging to cluster *c* (Figure 4A and Supplementary Figure 5). DSS treatment alone did not significantly affect the production of the lipids identified in cluster *c*. In cluster *b*, the concentrations of three lipids [resolvin D5 (RvD5), protectin DX (PDX), and 14-hydroxydocosahexaenoic acid (14-HdoHE)] were increased in the P + DSS group compared to the P group (Figures 4B–D). Only the PDx and 14-HdoHE concentrations were significantly increased in the P + DSS group compared to the DSS group (Figures 4C,D). Taken together, these data demonstrate that maternal prebiotic supplementation deeply modifies the basal lipid profile of the distal colon of mice and changes the production of 2 lipids under colitis conditions.

### Maternal prebiotic supplementation alters the gut microbiota composition and expression of microbiota-derived metabolites in offspring subjected to dextran sulphate sodium treatment

**FIGURE 5:**
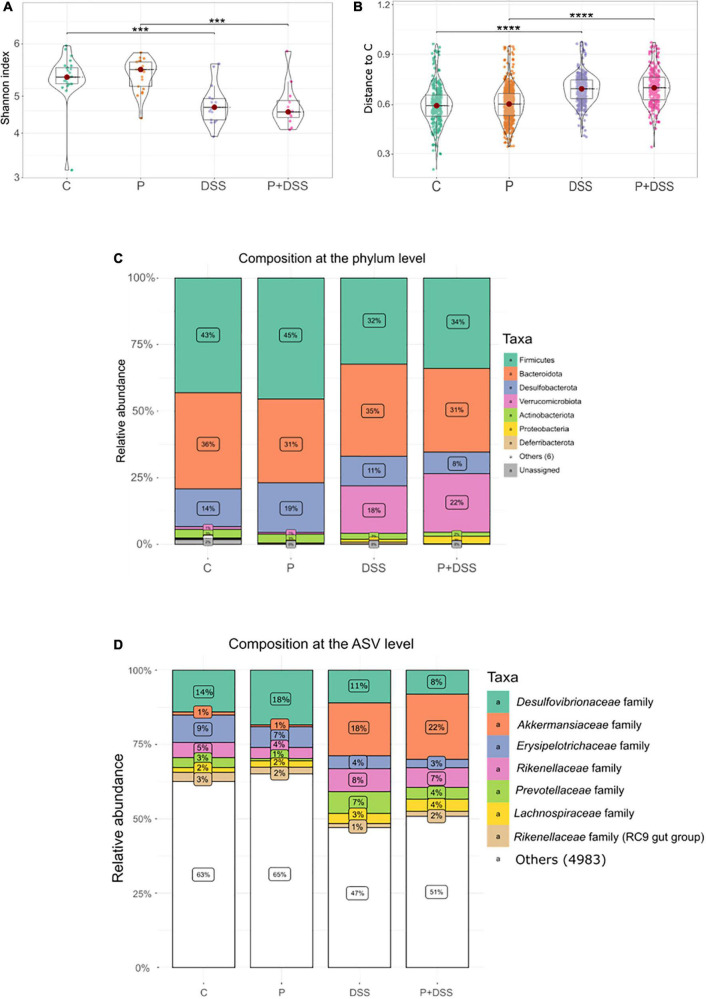
Dextran sulphate sodium (DSS) treatment decreases the α-diversity and alters the β-diversity independent of the maternal prebiotic supplementation that slightly changes the relative abundance of phyla. Impact of prebiotics on bacterial diversities was measured in the distal colons of all groups of mice. **(A)** Alpha diversity is represented by the Shannon index. **(B)** Distance from all samples to C samples was expressed as the Bray–Curtis dissimilarity. Relative abundance of bacterial populations at **(C)** phylum level and at **(D)** amplicon sequence variant (ASV) level with family classification. Kruskal–Wallis test followed by Dunn’s post-test **(A,B)**: ***p < 0.001, ****p < 0.001 (DSS effect). *N* = 12–24 mice/group at the beginning of the analysis.

Another focus of the study was to evaluate the extent to which maternal prebiotic supplementation could modify the composition and activity of the gut microbiota of the offspring under basal conditions and in response to DSS. First, the composition of microbiota in the luminal content of the distal colon was investigated. Alpha diversity, characterised by the Shannon index, decreased following DSS treatment, independent of maternal prebiotic supplementation (Figure 5A). Beta diversity analysis further emphasised the effects of DSS treatment. Distance tests (Bray–Curtis dissimilarities) showed that DSS-treated mice were more distant from the control group (Figure 5B and Supplementary Figure 6). PcoA (Supplementary Figure 6A) and PERMANOVA results (*p* = 0.0001) showed that DSS treatment led to a major shift in the microbial composition. The colonic bacterial composition was changed by DSS treatment at phylum and ASV levels, with an increase in the abundance of Verrucomicrobiota phylum, including *Akkermansia* genus of the *Akkermansiaceae* family, Proteobacteria phylum, *Alistipes* genus of the *Rikenellaceae* family, and *Alloprevotella* genus of the *Prevotellaceae* family, and a decrease in the Firmicutes phylum, *Desulfovibrionaceae* family, Desulfobacterota phylum, and *Faecalibaculum* genus of the *Erysipelotrichaceae* family (Figures 5C,D and Supplementary Figure 6B). This DSS-induced shift in bacterial abundance was confirmed by the Deseq2 analysis (Supplementary Figures 6C,D).

**FIGURE 6:**
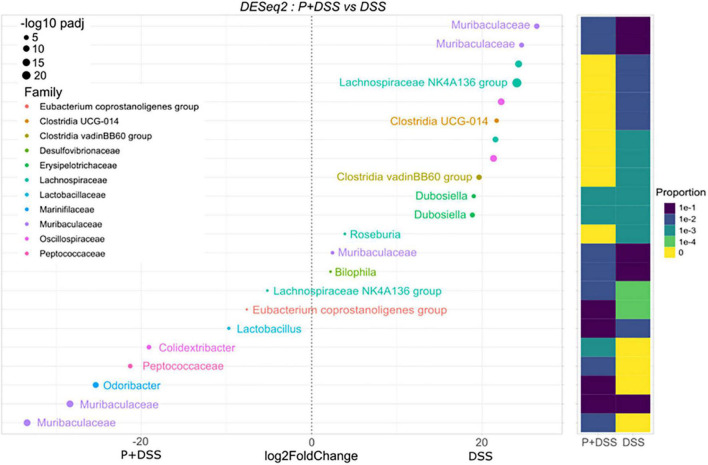
Prebiotic administration to mothers changes the relative abundance of several bacterial genera in the offspring treated with DSS. Impact of prebiotic supplementation on the bacterial composition was analysed in the luminal content of the distal colons of all groups of mice by DESeq2, which shows differentially abundant ASVs between P + DSS *vs.* DSS groups of mice. *N* = 12–24 mice/group at the beginning of the analysis.

Regarding the effect of maternal prebiotic supplementation in the DSS condition on colonic bacterial composition, eight ASVs were found to be overabundant in the P + DSS group compared to the DSS group, with two ASVs belonging to the *Muribaculaceae* family, three ASVs of the genera *Odoribacter*, *Colidextribacter*, and *Lactobacillus*, and three ASVs of the *Peptococcaceae* family, *Eubacterium coprostanoligenes* group, and *Lachnospiraceae* NK4A136 group (Figure 6). Ten ASVs were found to be less abundant in the P + DSS group than in the DSS group. These 10 ASVs belonged to the *Muribaculaceae, Lachnospiraceae, Oscillospiraceae*, *Clostridia*, *Dubossiella*, *Bilophila*, and *Roseburia* genera (Figure 6). In addition to these differences under DSS treatment, the basal gut bacterial composition between the P and C groups also differed, with ASVs assigned to the *Muribaculaceae* and *Lachnospiraceae* families being overabundant in the P group (Supplementary Figure 6E). Taken together, maternal prebiotic supplementation induced remodelling of the gut bacterial composition, with or without DSS treatment.

**FIGURE 7:**
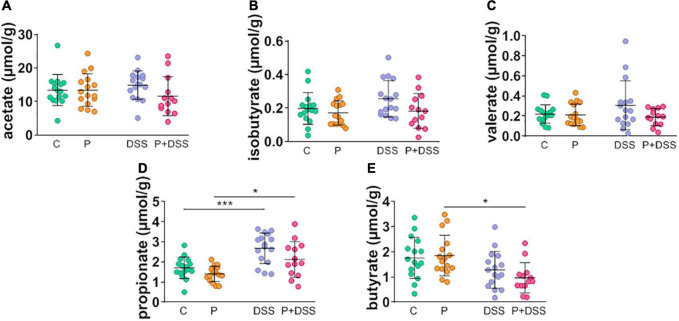
Prebiotic administration to mothers decreases the caecal short-chain fatty acid butyrate concentration in offspring treated with DSS. **(A–E)** Impact of prebiotic supplementation and/or DSS treatment on the concentrations of different SCFAs was measured in the caecal content of all groups of mice. Data represent the mean and standard deviation of the mean of 11–17 mice per group. Two-way ANOVA, followed by Bonferroni’s *post hoc* comparisons tests: *p < 0.05, ***p < 0.001 (Prebiotics or DSS effect).

Next, we aimed to determine whether changes in bacterial composition were associated with changes in the production of SCFAs by assessing their concentrations in the caecum (Figures 7A–E). No significant modification of acetate, isobutyrate, or valerate concentrations was observed under any conditions (Figures 7A–C). DSS induced an increase in propionate concentration that was not affected by maternal prebiotic supplementation (Figure 7D). SCFA concentrations were not significantly different between the DSS and P + DSS groups (Figures 7A–E). Interestingly, butyrate concentration was significantly lower in the P + DSS group than in the control group (Figure 7E). These results suggest that maternal prebiotic supplementation induces changes in the gut microbiota composition and activity in the offspring, with and without chronic colitis.

## Discussion

The present study tested the hypothesis that similar to the observed reduction in the occurrence of food allergy, asthma, and skin inflammation (9, 45–47), maternal prebiotic supplementation can prevent or reduce the development of colitis in their offspring. This presumptive protective effect is in fact absent, and certain parameters reflecting colitis seem even aggravated in pups from mothers exposed to prebiotics during gestation. This susceptibility was represented by transient changes in weight loss and transit time, an increase in faecal humidity, changes in distal colonic lipid content, and alteration of the distal colonic luminal microbiota.

Apart from its impact on the response to DSS and development of colitis, our study showed the major imprint of the mother’s diet on healthy offspring. Indeed, healthy offspring from mothers supplemented with the prebiotics, GOSs and inulin, presented a shortened colon, changes in distal colonic luminal microbiota composition, and changes in distal colonic transcriptomic and lipidomic profiles. This study did not determine any imprinting mechanisms, as two studies have already addressed this subject. These studies were conducted in parallel to our work and shared our study mice mothers, with a part of the protocol comprising the breeding to weaning periods (6, 10). Parallel studies have demonstrated that maternal supplementation with GOSs and inulin did not affect the gestational outcomes, such as maternal weight at 18 d of gestation or the average number of pups per litre (6). However, they underlined the effects of GOSs and inulin on mouse mothers, *in utero* effects on the foetus, and effects later in the life of the offspring (6, 10). Maternal prebiotic supplementation induced a modification of the gut microbiota of mice mothers by increasing the relative abundance of Bacteroidetes and Proteobacteria phyla, decreasing the abundance of Desulfobacteria and Firmicutes phyla, and modulating ASV abundances assigned to *Muribaculaceae* family (6). This modification was associated with an increase in the production of SCFAs, especially acetate and propionate (6), in stools, which was maintained until the mid-lactation period (10). This maternal prebiotic supplementation also changed the gut microbiota composition and gut metabolite production of the 6-weeks-old female offspring. In comparison to the mice mothers, a different profile was observed in the female offspring, with changes in the colonic content of propionate and butyrate (not in faeces), increased relative abundance of *Bifidobacteriaceae*, *Desulfovibrionaceae*, and *Sutterellaceae* families, and five identified ASVs from *Rikenellaceae*, *Atopobiaceae*, *Bacteroidaceae*, and *Lachnospiraceae* families in stools (10). This profile was not found in our 11-weeks-old male pups from prebiotic-supplemented mothers, which may be explained by differences between the studies in terms of age, sex, and sampling site (luminal content, tissue, or stool). Nevertheless, both studies in offspring have shown that their gut microbiota composition was different from that of control pups and presented distinct ASVs mainly assigned to the *Muribaculaceae* family (Supplementary Figure 5). In addition to the imprint on the gut microbiota, an impact on host transcripts was observed, with changes in the expression of genes involved in cell maturation and villi development. Indeed, the identified genes were associated with spermatogenesis, but spermatogenesis was better documented than villi development. Therefore, we can assume that the genes assigned to sperm capacitation or motility are concerned with the formation and functionality of the villi, as they require the same molecular factors (48). On a different scale, colon shortening was observed in our study and could either reflect colonic inflammation in mouse models (26) or result from smooth muscle contraction induced by prostaglandins (49), such as PGE_2_ and PGF_2α_ . Interestingly, the concentrations of these two lipids, along with 26 other lipids, were increased in the colon of offspring from prebiotics-supplemented mothers (Figure 4 and Supplementary Figure 4). These mediators are involved in numerous functions, such as protection against gastric erosion (50). An increasing number of studies have described the regulation of epithelial apoptosis (26), healing (16), and permeability (23). Their classification into pro-inflammatory (PGE_2_, PGE_3_, LTB_4_, and LTB_5_) and pro-resolutive (RvD1 and LxA4) mediators (38) is insufficient to explain their roles in the intestine (51), especially considering that 3-hydroxylated long-chain fatty acids (C12-, C14-, C16-, and C18-3OH) can be produced by bacteria and protect against colitis (52). The impact of these oxylipins on IBD could be greater than expected, as they can be regulated by diet and/or induce microbiota changes (53, 54).

In this study, we investigated how this imprint could precondition an inappropriate response to a noxious stimulus, such as DSS, and which mechanisms were responsible for the enhancement of some parameters reflecting colitis in the offspring of prebiotic-supplemented mice. Indeed, if colitis is not massively increased (no significant changes of the survival curves, the weight loss or the intestinal transit at day 18, the micro-DAI, the Lcn-2 faecal concentration or the global gene expression) the P + DSS group showed some exacerbated responses to DSS (transient changes in weight loss and transit time, an increase in DAI and faecal humidity) *via* modification of the distal colonic luminal microbiota composition and changes in distal colonic lipid content. To date, colitis prevention studies have mainly been performed in adult murine models, and while they showed, for the most part, protective effects (55–58), two studies described how inulin supplementation potentiated the severity of colitis (59, 60). Recently, in line with our work, one study analysed the effect of maternal intake of inulin on colitis development in rats and described how it exacerbated DSS-induced intestinal damage and inflammation (61). The increased DAI, myeloperoxidase activity, and IL-1β mRNA expression observed in this model are associated with an increase in the abundance of Bacteroidetes, Bacteroides, and Parasutterella phyla (61). In our study, the gut microbiota from the P + DSS group presented a reshaping of the *Muribaculaceae* and *Lacnospiraceae* groups, along with specific overabundant *Peptococcaceae*, *Odoribacter*, *Colidextribacter*, *Lactobacillus*, and *Eubacterium coprostanoligenes*, and a reduced representation of *Dubossiella, Clostridia*, *Bilophila*, and *Roseburia.* Among these bacteria, some are associated with colitis protection, whereas others have been assigned as contributors to colitis. Indeed, a reduction in *Odoribacter* and *Lactobacillus* abundance is observed in IBD (62, 63). *Odoribacter* strains have beneficial effects associated with their ability to produce SCFAs or induce IL-10 in a mouse colitis model (62). Similarly, *Lactobacillus* strains have been used as probiotics to alleviate IBD (63). Some of these strains ameliorate colitis in mice and rats and reduce the expression of TNF-α and IL-17 in UC patients biopsies (64, 65). *Eubacterium coprostanoligenes* also ameliorates colitis development in fibre-free diet-fed mice (66). Therefore, these strains could not explain the increased colitis observed in P + DSS animals, whereas *Maribaculaceae* and *Lacnospiraceae* could. Indeed, a decrease in *Muribaculaceae* and *Lachnospiraceae_ raceae_NK4A136_*group abundance has been shown to contribute to colitis development in mice (67). In addition, the decrease in butyrate production observed in the P + DSS group supports the deleterious effect of maternal prebiotic consumption on colitis development. Butyrate is an SCFA that contributes to the gut barrier through its primary role as an energy source for colonocytes, promotion of IEC differentiation, production of antimicrobial peptides, and modulation of tight-junction proteins (14, 68). Butyrate also has anti-inflammatory properties mediated by epigenetic mechanisms and its ability to restore Treg/Th17 balance (68, 69). These changes in the microbiota were accompanied by changes in the production of 3-hydroxylated long-chain fatty acids, and the observed increase in the P group was completely lost in the P + DSS group (Figure 4 and Supplementary Figure 4). One of these mediators, 3-hydroxyoctadecaenoic acid (C18-3OH), has anti-inflammatory effects and may protect against DSS-induced colitis, as its production is abolished by DSS (52).

Changes induced in the offspring of prebiotic-supplemented mice were not limited to the microbiota. Although T cell subpopulations were not analysed, an increase in T cell and macrophage infiltration was observed in the distal colon, in accordance with the DSS model which features an upregulation of T cells and macrophages (70). This upregulation of the immune response by maternal prebiotic supplementation may even imply a persistent aspect of inflammation; persistence of inflammatory signals is known as a main feature of chronic inflammation (71). Compared to a study on food allergy which demonstrated the protective effects of the same prebiotics used in our protocol (10), our disease model could involve a different immune response. Indeed, the immune response in food allergy is specific to an allergen and involves Th2 cells; in our DSS-induced chronic colitis model, the inflammatory response was induced by a chemical linked to Th1 and Th17 cell activation (11, 70). The difference in the effects of maternal prebiotic supplementation on colitis and food allergies could also be explained by discrepancies in the design of the protocol. Indeed, experiments have been conducted on animals of different genders and age (72). Gender may have an effect on colitis development and female mice tend to be more resistant to colitis induction (72). We therefore decided to focus first our study on 8–10 weeks old males to avoid gender effect while the food allergy study used 6 weeks old female mice (9, 10). Associated with our gene expression analysis, we could argue that our DSS model is Th17-dependent and that maternal prebiotic supplementation did not modify the Th17 immune profile, as characterised by an increased IL-17 signalling pathway in both DSS and P + DSS groups. However, the identified genes *Cxcl1, Tnfrsf1b*, and *Tnfrsf23* in the P + DSS condition were associated with modulation of gut inflammation. CXCL1 is involved in neutrophil recruitment during inflammation in IBD patients and is secreted by macrophages (73). *Tnfrsf1b* codes for TNFR2, a receptor expressed on T regulatory cells that is involved in their expansion, proliferation, suppressive function, and tissue regeneration (74, 75). *Tnfrsf23* participates in T cell apoptosis and inhibits colonic CD8 + T cells expansion (76).

Transcriptomic analysis revealed that, in addition to cytokine signalling, the regulated genes were linked to bioactive lipid metabolism, which could also participate in immune regulation. The downregulated *Hpgds* gene encodes the enzyme that produces prostaglandin D2 (PGD_2_), one of the lipids secreted by the mouse macrophage cell line RAW 264.7 upon TLR4 binding (77, 78). The role of PGD_2_ remains debated since Hokari et al. suggested that L-PGDS plays a pro-inflammatory role in the development of colitis in clinical and experimental studies (79). In contrast, PGD_2_/DP1 axis activation has been shown to confer anti-inflammatory properties (80) and reduce colitis development (81). Among the other host distal colonic lipid concentrations affected, an increase in RvD5, 14-HdoHE, and PDX concentrations was observed only in the P + DSS group. These lipids are derived from *n* −3 PUFA docosahexaenoic acid (DHA) via a lipoxygenase-mediated synthesis pathway (82, 83). It is difficult to link these modifications to increased colitis development because RvD5 reduced DSS-colitis inflammation in mice (71), and 14-HdoHE and PDX are mainly described as anti-inflammatory mediators (84, 85). Similar to the increased RvD5 production observed in the P + DSS condition, the resolving pathway was upregulated in human IBD colon biopsies (71). PDX also exhibits anti-inflammatory properties through the inhibition of cyclooxygenase (COX)-1 and COX2, and through the reduction of reactive oxygen species production in human blood neutrophils (82). Nevertheless, its inhibition of COX1 and COX2 could have deleterious effects on the intestinal epithelial barrier and could be linked to exacerbations in patients with IBD (86).

In addition to the increased infiltration of T cells and macrophages in the distal colon and microbiota remodelling, the observed transcriptomic changes helped us to better understand the broad impact of maternal prebiotic supplementation on colitis development in the offspring. For example, the downregulation of *Aqp11* observed in the P + DSS group suggests that maternal prebiotic supplementation modified the offspring’s endoplasmic reticulum (ER) sensitivity to stress and redox signalling. Unlike other peroxiporins, AQP11 is localised in the ER. Its downregulation strongly disrupts the flux of hydrogen peroxide (87). ER stress has been observed in IBD (88). In addition to the modules related to inflammation, lipid metabolism, and epithelial functions, computational transcriptomic analysis revealed genes related to neurological processes, including connectivity (*Sema5a*), channels (*Kcna1*, *Cacna1d*, and *Kcnk6*), neurotransmitters (*VIP*), neurotransmitter production (*Comt*, *Maoa*, and *Maob*), and tryptophan metabolism (*Maoa* and *Maob*). *Comt* and *Maoa* are both specifically downregulated in the P + DSS group and encode catecholamine degradation enzymes (89, 90). Catecholamines are important for gut function, including nutrient absorption and gut motility, and interaction with the immune system (91). Even though MAO inhibitors exert anti-inflammatory effects (92), they are similar to COMT inhibition (93) and intestinal adverse effects (nausea and vomiting). Therefore, we investigated the extent to which an alteration in the concentration of gut catecholamines may contribute to the impact of maternal prebiotic consumption on intestinal permeability.

Altogether, our results showed that maternal prebiotic supplementation had a major imprint on offspring that persisted in the adult stage. These long-term effects were insufficient to prevent colitis and contributed to worsen some parameters of its development. Further investigation is necessary to rule out the pro-inflammatory or pro-resolving nature of the persistent immune response observed in the distal colon of DSS-treated offspring from prebiotic-supplemented mothers. Our work raises questions about the potentially harmful effects of prebiotics, which are supposed to benefit the host by definition. Our study also underlines the specificity of each chronic disease, which may facilitate future research on the nutritional modulation of gut microbiota for IBD care and prevention.

## Data availability statement

The data presented in this study are deposited in the ArrayExpress repository, accession number E-MTAB-12176; and ENA repository, accession number PRJEB55683.

## Ethics statement

The animal study was reviewed and approved by Comité d’Ethique en Expérimentation Animale des Pays-de-la-Loire (accession number: 16925).

## Author contributions

AL, MR-D, MN, and MB: study conceptualization. AL and MR-D: formal analysis and writing – original draft preparation. AL, ED, PB, and MR-D: statistics. MR-D, MN, and MB: resources and funding acquisition. AL, MR-D, MB, and MN: writing – review and editing. MR-D and MB: supervision. MR-D, MB, and MN: project administration. All authors contributed to the article and approved the submitted version.

## Footnotes

1
https://www.metaboanalyst.ca/

2
https://gitlab.univ-nantes.fr/bird_pipeline_registry/microSysMics

## Supplementary material

Supplementary Figure 1 Table of the 53 lipids quantified in the mouse colons using liquid chromatography-tandem mass spectrometry analysis.

Supplementary Figure 2 Maternal prebiotic supplementation increases the disease activity index at day 18 and total transit time at day 4 in offspring, but does not change at day 18 the DSS-induced decrease in distal intestinal motility and protects from DSS-induced increase in permeabilities. Impact of prebiotics on colitis severity and intestinal functions was measured in all groups of mice during the protocol through DAI **(A)**, motility **(B–D)** and permeability evaluations **(E,F)**. **(A)** Disease activity index (DAI) of each mouse. *N* = 20–28 mice/group. Motility was evaluated using TTT **(B,D)** and FPO. **(C)** *N* = 17–28 and 19–28 mice/group, respectively. **(E)** *In vivo* paracellular permeability was evaluated by FSA flow measurement. *N* = 19–28 mice/group. **(F)** *Ex vivo* transcellular permeability in proximal colon was characterised by the area under curve of HRP mean flow in Ussing chamber. *N* = 17–27 mice/group. Data represent the mean and standard deviation. Two-way ANOVA, followed by Bonferroni’s *post hoc* comparisons tests: **p* < 0.05, ^**^*p* < 0.01, ^***^*p* < 0.001, ^****^*p* < 0.0001 (Prebiotics or DSS effect).

Supplementary Figure 3 Dextran sulphate sodium (DSS) treatment differentially changes the transcriptomic profile of the distal colon of offspring depending on the maternal diet: Standard or prebiotic-supplemented. Impact of DSS treatment on gene expression was measured in the distal colon of the DSS *vs.* C and P + DSS *vs.* P groups. Differentially expressed genes (upregulated in bold text for DSS and P + DSS groups and upregulated in plain grey text for C and P groups) were associated with several KEGG clusters. **(A,B)** Tables of the nine most relevant KEGG clusters in both comparisons of groups. Statistics were generated using clusterProfiler 3.14. Specific and common genes associated to P as the response to DSS treatment (*cf*. Figure 3C) were represented in a Venn diagram **(C)** and a detailed table **(D)** with genes written in bold text.

Supplementary Figure 4 Prebiotic maternal supplementation changes the transcriptomic profile of the distal colon of offspring in basal condition. Basal impact of maternal prebiotics on gene expression was measured in the distal colon via the comparison of groups C and P of mice. **(A)** Volcano plot showing differentially expressed genes in the comparison of groups P and C. Genes upregulated in the P or C group are represented in orange or green, respectively. **(B,C)** Tables of 10 and 4 most relevant GO and KEGG clusters, respectively, in the comparison of groups P and C with upregulated genes of the P group written in bold text. Statistics were generated using clusterProfiler 3.14.

Supplementary Figure 5 Prebiotic administration to mothers changes the concentrations of 28 lipids in the distal colon. In the heatmap showing the mean normalised concentrations of 49 lipid mediators in distal colon for each group of mice, a cluster of 28 metabolites with similar concentration variation was identified in the P group. The levels of lipids written in bold text were significantly decreased in the P + DSS *vs*. P group. Data represent the adjusted *p*-values and ratio of the mean concentration for each comparison of groups. *N* = 12–16 mice/group. Two-way ANOVA, followed by Bonferroni’s *post hoc* comparisons tests. N/A: not applicable.

Supplementary Figure 6 Maternal prebiotics increase the levels of seven ASVs, including four belonging to *Muribaculaceae*, in offspring in the basal condition. **(A)** Principal coordinate analysis (PCoA) using the Bray–Curtis dissimilarity index. **(B)** ASV names and their most detailed classification found in Figure 5D, which represents the relative abundance of bacterial populations at ASV level. Impact of DSS treatment on the bacterial composition was analysed in the luminal content of the distal colons of all groups of mice by DESeq2, which showed differentially abundant ASVs between **(C)** DSS *vs*. C and **(D)** P + DSS *vs*. P groups of mice. Basal impact of maternal prebiotics on the bacterial composition was analysed in the distal colonic luminal content of the P *vs.* C group of mice by DESeq2. **(E)** Differentially abundant ASVs between P and C groups of mice.
