# Supplementary figures and images for: Maternal prebiotic supplementation impacts colitis development in offspring mice
Source: Front Nutr. 2023 Jan 5;9:988529. doi: 10.3389/fnut.2022.988529 (PMC9849907; doi:10.3389/fnut.2022.988529)

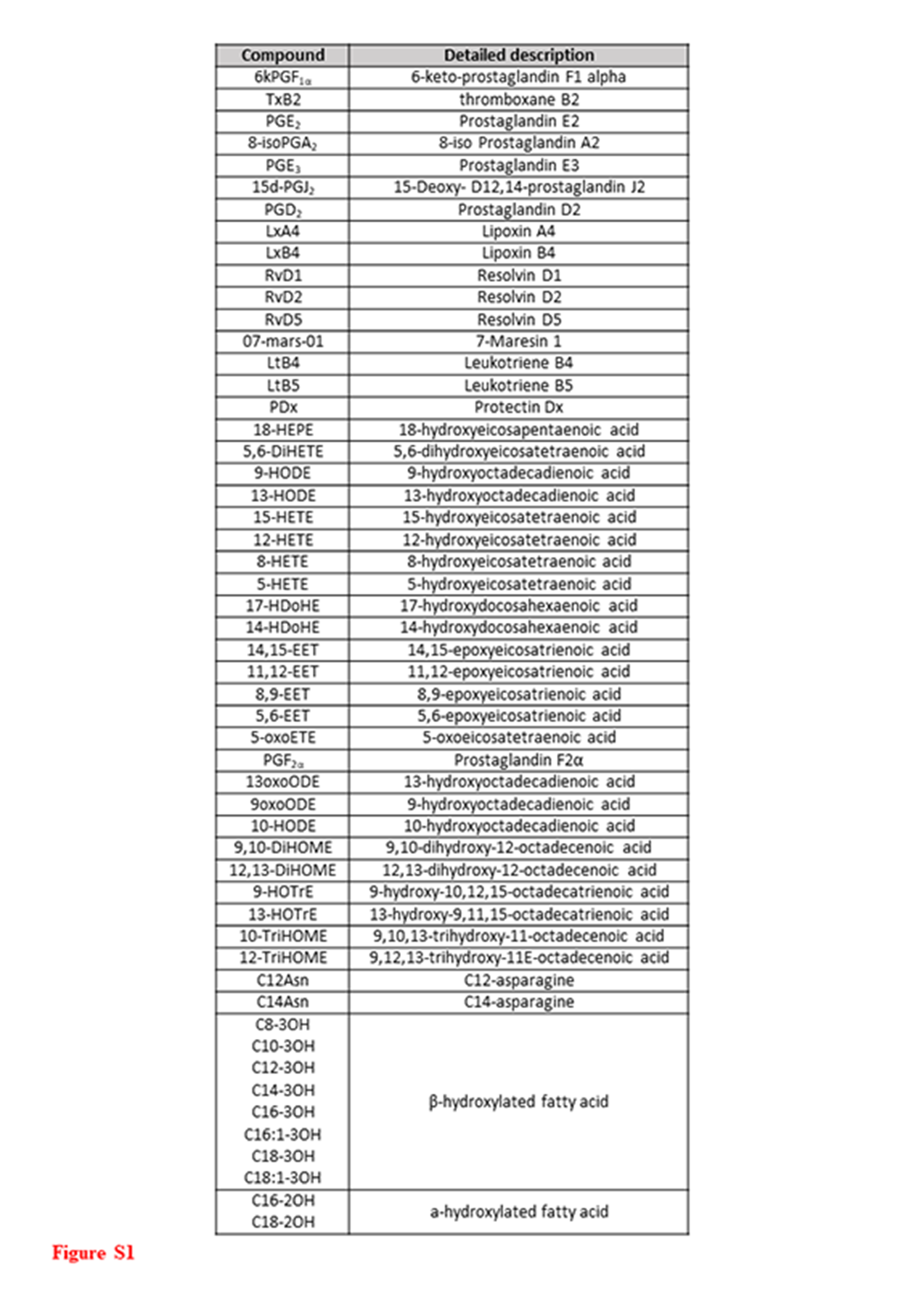

Supplement: Supplementary Figure 1 — Table of the 53 lipids quantified in the mouse colons using liquid chromatography-tandem mass spectrometry analysis. [file Image_1.TIF]

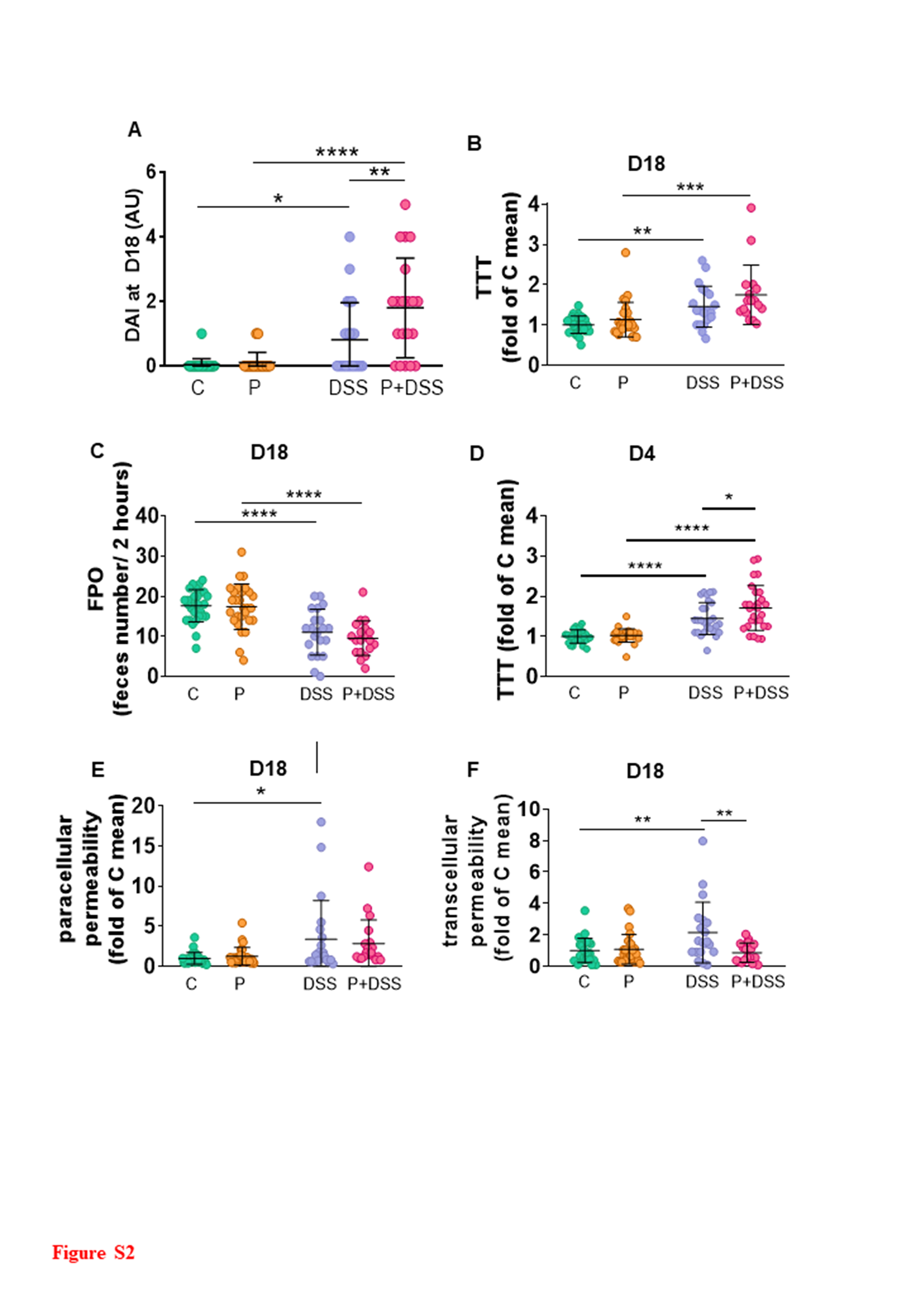

Supplement: Supplementary Figure 2 — Maternal prebiotic supplementation increases the disease activity index at day 18 and total transit time at day 4 in offspring, but does not change at day 18 the DSS-induced decrease in distal intestinal motility and protects from DSS-induced increase in permeabilities. Impact of prebiotics on colitis severity and intestinal functions was measured in all groups of mice during the protocol through DAI (A), motility (B–D) and permeability evaluations (E,F). (A) Disease activity index (DAI) of each mouse. N = 20–28 mice/group. Motility was evaluated using TTT (B,D) and FPO. (C) N = 17–28 and 19–28 mice/group, respectively. (E) In vivo paracellular permeability was evaluated by FSA flow measurement. N = 19–28 mice/group. (F) Ex vivo transcellular permeability in proximal colon was characterised by the area under curve of HRP mean flow in Ussing chamber. N = 17–27 mice/group. Data represent the mean and standard deviation. Two-way ANOVA, followed by Bonferroni’s post hoc comparisons tests: *p < 0.05, **p < 0.01, ***p < 0.001, ****p < 0.0001 (Prebiotics or DSS effect). [file Image_2.TIF]

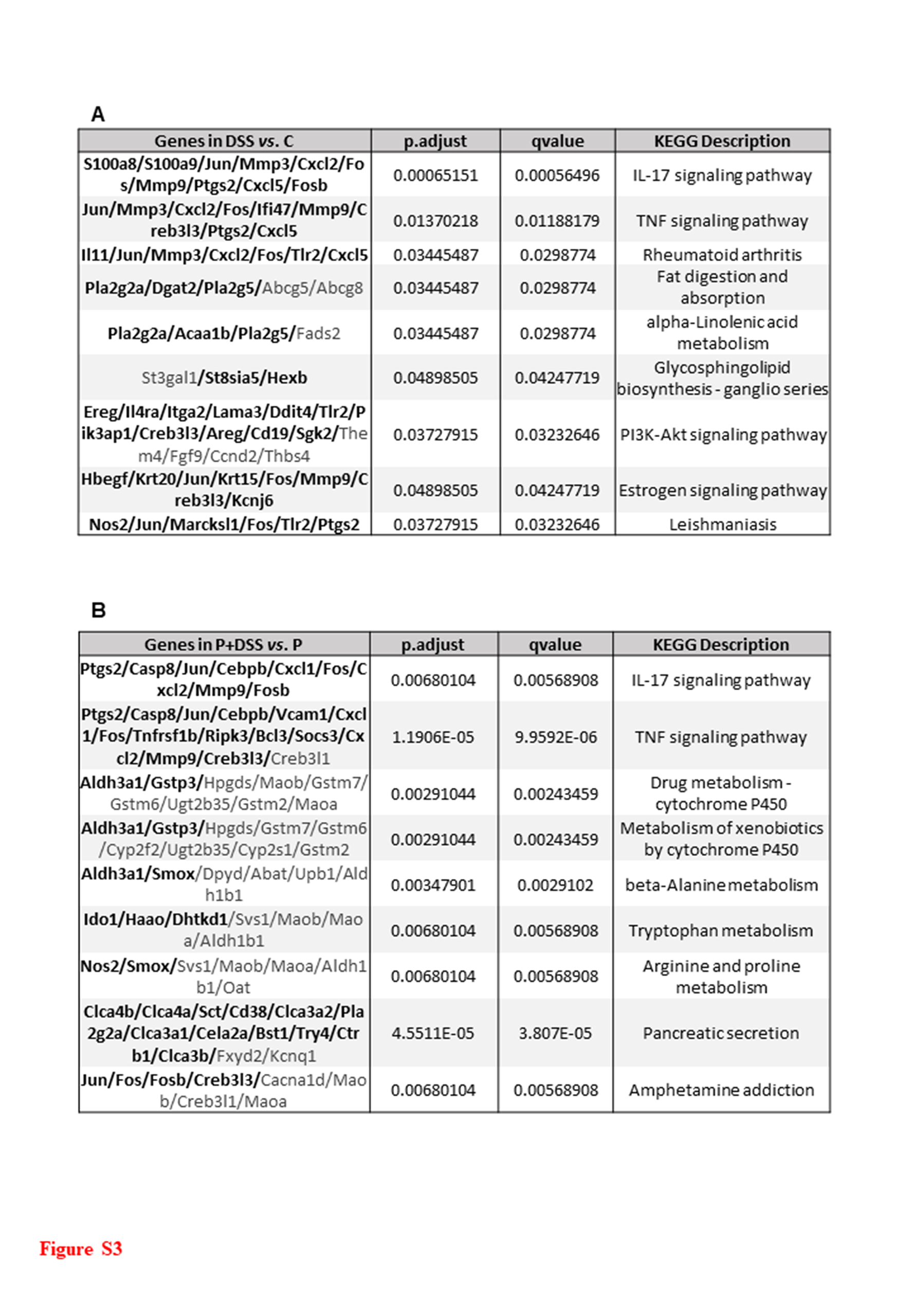

Supplement: Supplementary Figure 3 — Dextran sulphate sodium (DSS) treatment differentially changes the transcriptomic profile of the distal colon of offspring depending on the maternal diet: Standard or prebiotic-supplemented. Impact of DSS treatment on gene expression was measured in the distal colon of the DSS vs. C and P + DSS vs. P groups. Differentially expressed genes (upregulated in bold text for DSS and P + DSS groups and upregulated in plain grey text for C and P groups) were associated with several KEGG clusters. (A,B) Tables of the nine most relevant KEGG clusters in both comparisons of groups. Statistics were generated using clusterProfiler 3.14. Specific and common genes associated to P as the response to DSS treatment (cf. Figure 3C) were represented in a Venn diagram (C) and a detailed table (D) with genes written in bold text. [file Image_3.TIF]

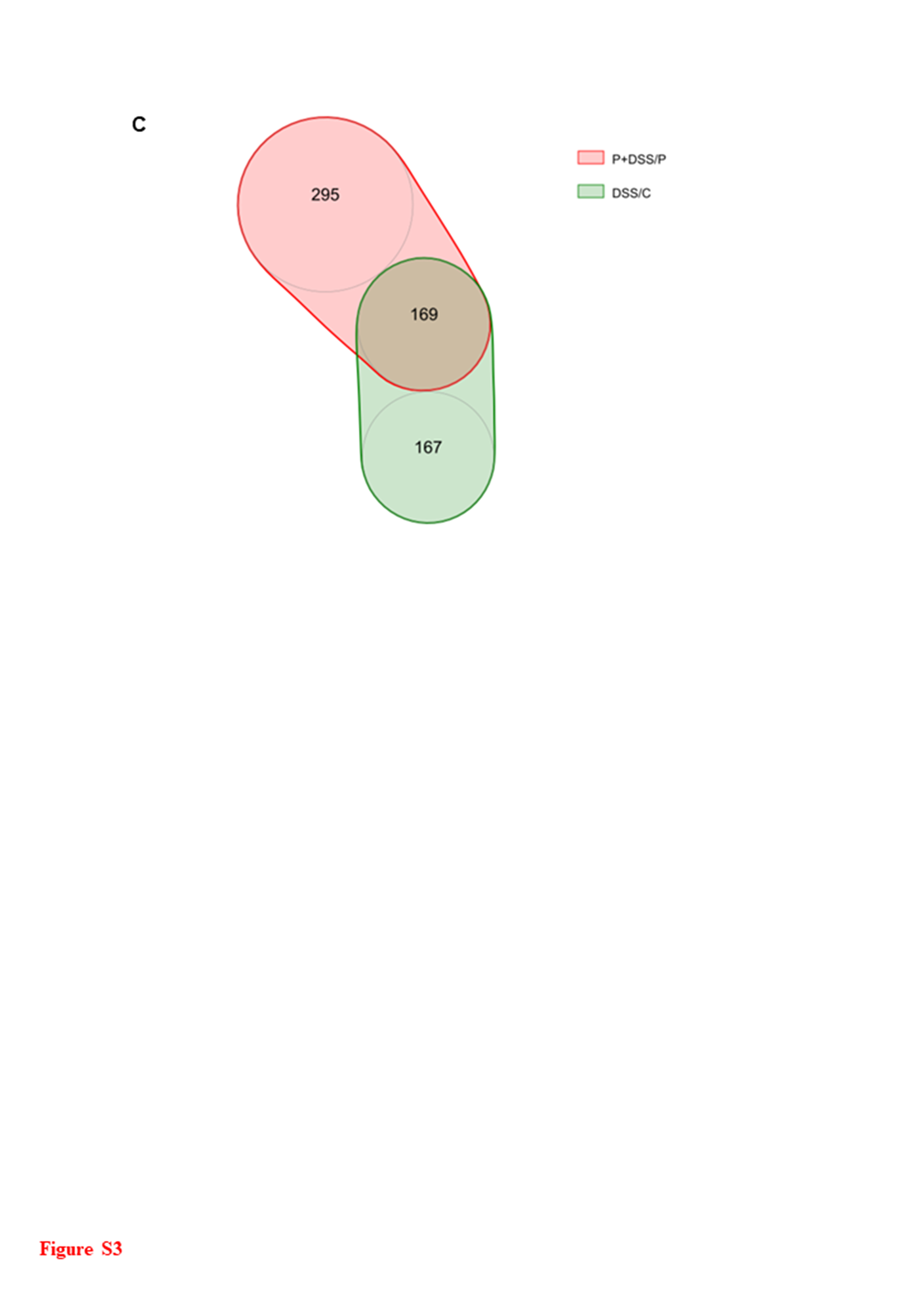

Supplement: Supplementary file 4 [file Image_4.TIF]

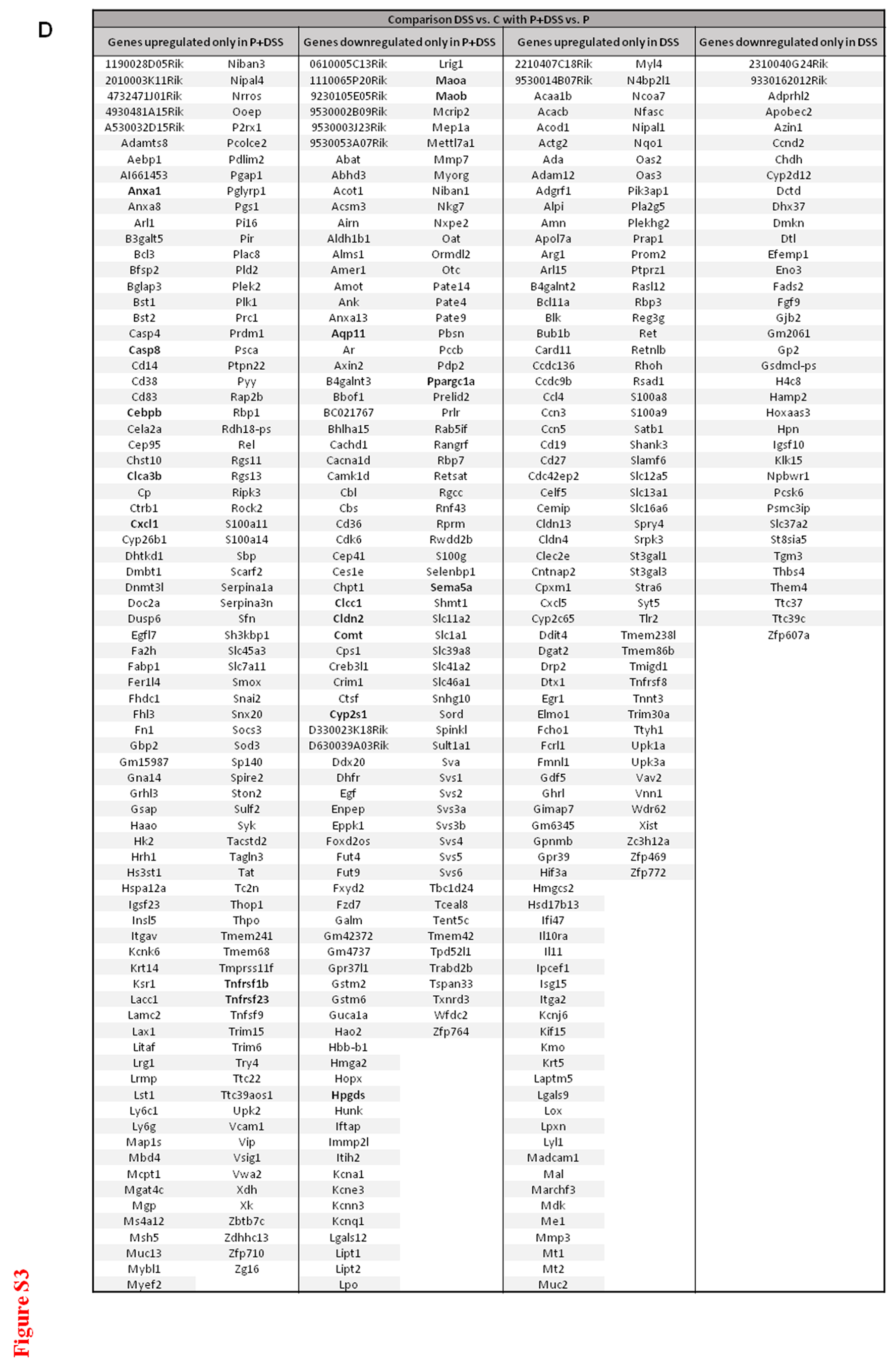

Supplement: Supplementary file 5 [file Image_5.tif]

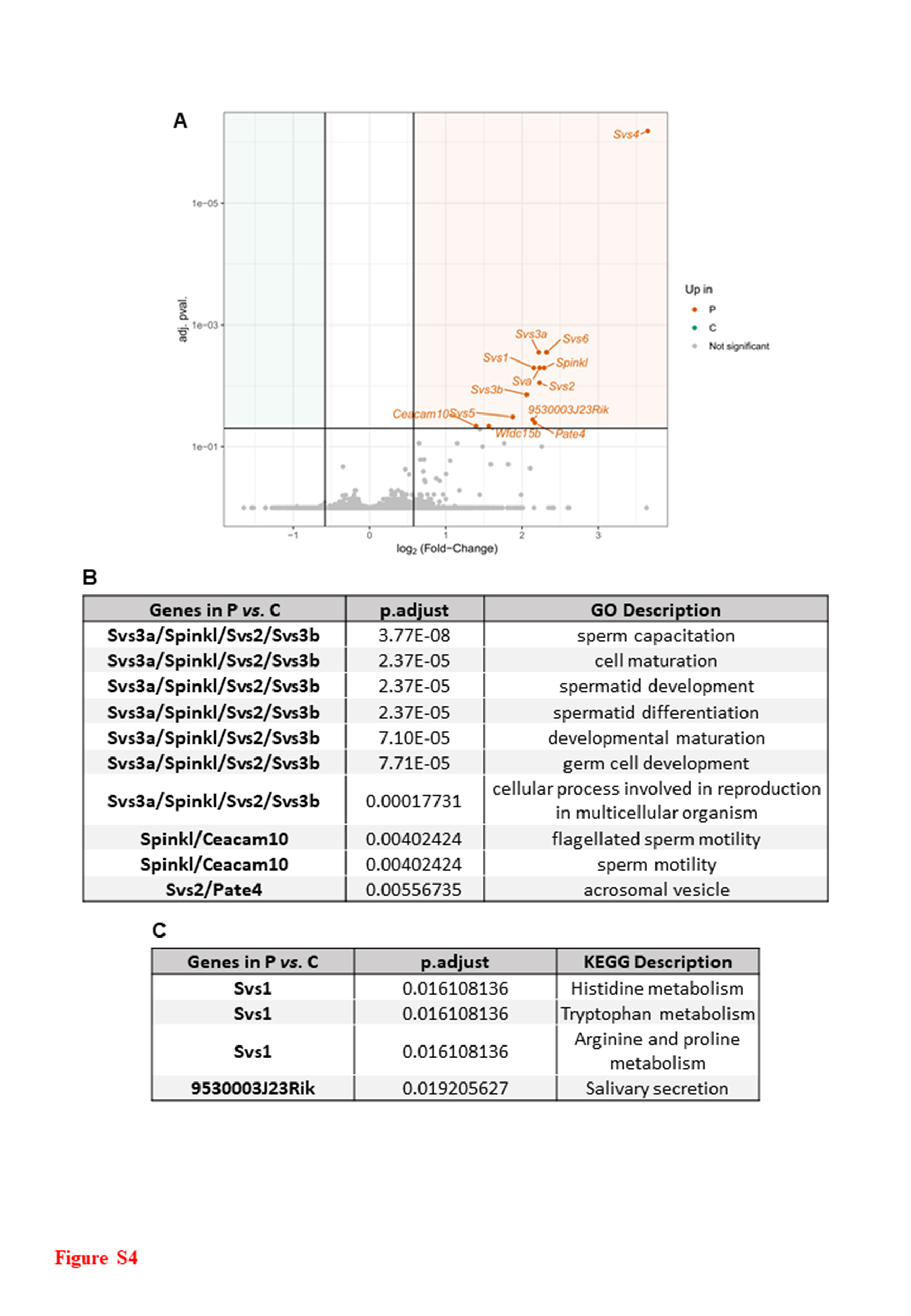

Supplement: Supplementary Figure 4 — Prebiotic maternal supplementation changes the transcriptomic profile of the distal colon of offspring in basal condition. Basal impact of maternal prebiotics on gene expression was measured in the distal colon via the comparison of groups C and P of mice. (A) Volcano plot showing differentially expressed genes in the comparison of groups P and C. Genes upregulated in the P or C group are represented in orange or green, respectively. (B,C) Tables of 10 and 4 most relevant GO and KEGG clusters, respectively, in the comparison of groups P and C with upregulated genes of the P group written in bold text. Statistics were generated using clusterProfiler 3.14. [file Image_6.TIF]

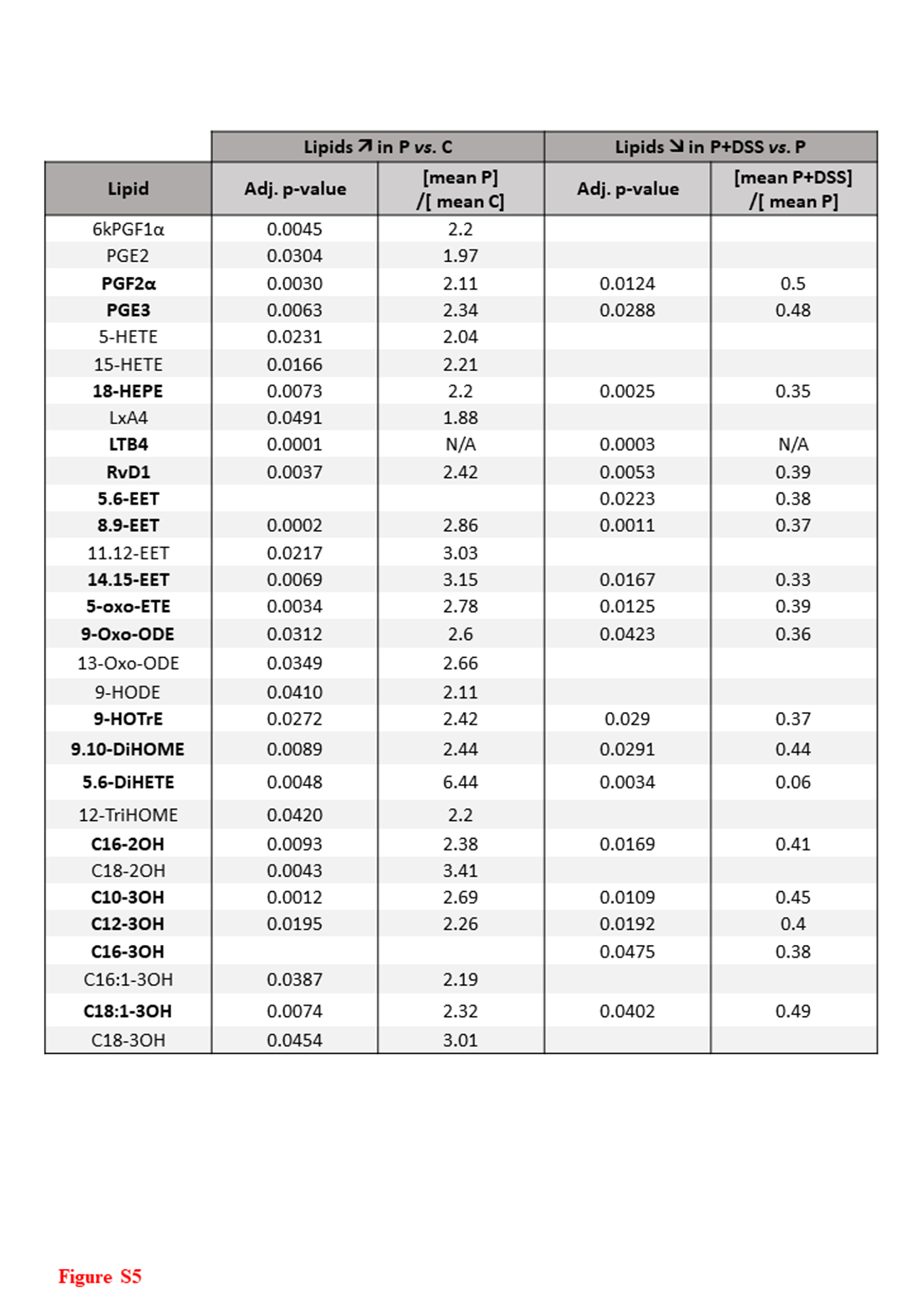

Supplement: Supplementary Figure 5 — Prebiotic administration to mothers changes the concentrations of 28 lipids in the distal colon. In the heatmap showing the mean normalised concentrations of 49 lipid mediators in distal colon for each group of mice, a cluster of 28 metabolites with similar concentration variation was identified in the P group. The levels of lipids written in bold text were significantly decreased in the P + DSS vs. P group. Data represent the adjusted p-values and ratio of the mean concentration for each comparison of groups. N = 12–16 mice/group. Two-way ANOVA, followed by Bonferroni’s post hoc comparisons tests. N/A: not applicable. [file Image_7.TIF]

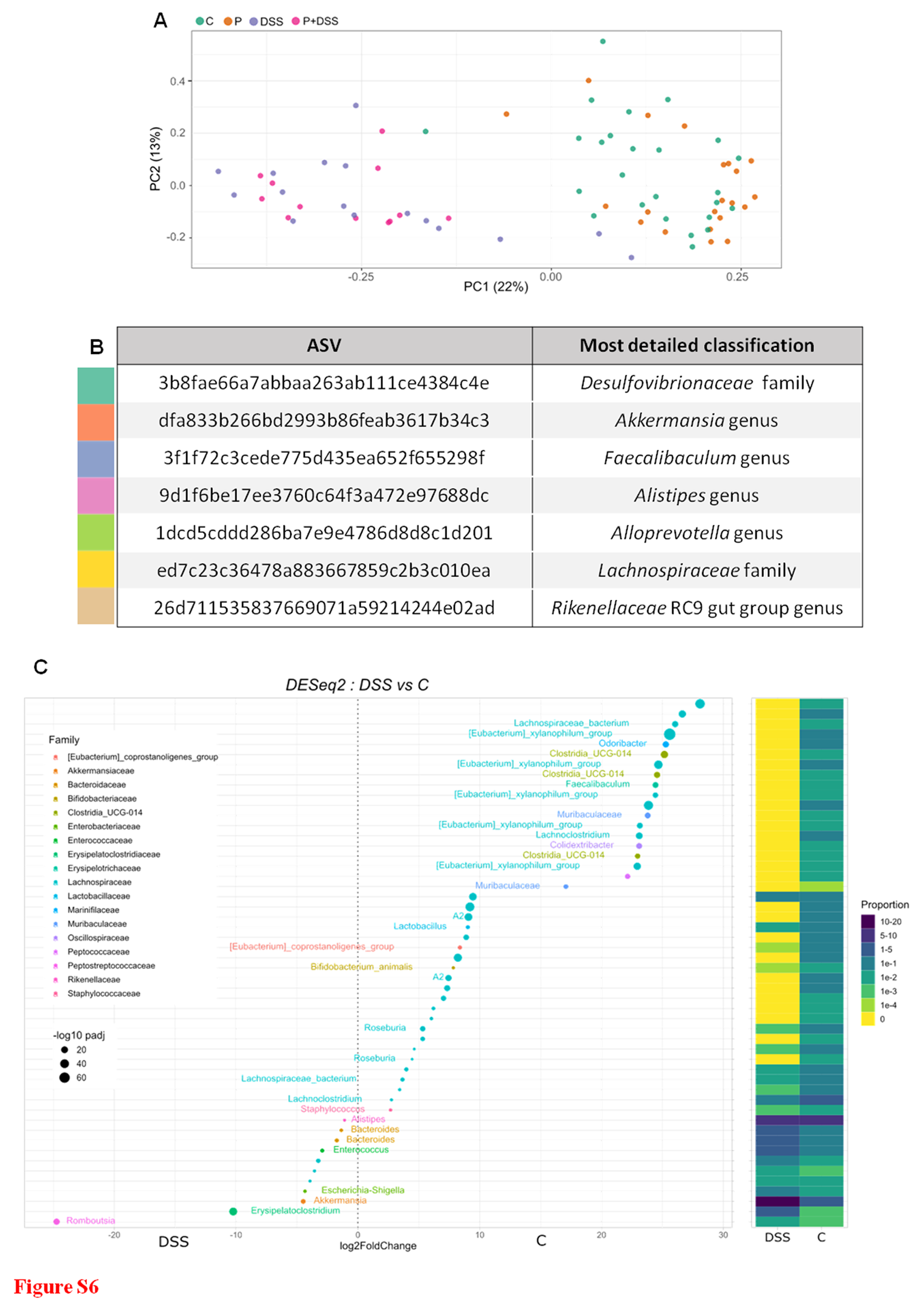

Supplement: Supplementary Figure 6 — Maternal prebiotics increase the levels of seven ASVs, including four belonging to Muribaculaceae, in offspring in the basal condition. (A) Principal coordinate analysis (PCoA) using the Bray–Curtis dissimilarity index. (B) ASV names and their most detailed classification found in Figure 5D, which represents the relative abundance of bacterial populations at ASV level. Impact of DSS treatment on the bacterial composition was analysed in the luminal content of the distal colons of all groups of mice by DESeq2, which showed differentially abundant ASVs between (C) DSS vs. C and (D) P + DSS vs. P groups of mice. Basal impact of maternal prebiotics on the bacterial composition was analysed in the distal colonic luminal content of the P vs. C group of mice by DESeq2. (E) Differentially abundant ASVs between P and C groups of mice. [file Image_8.tif]

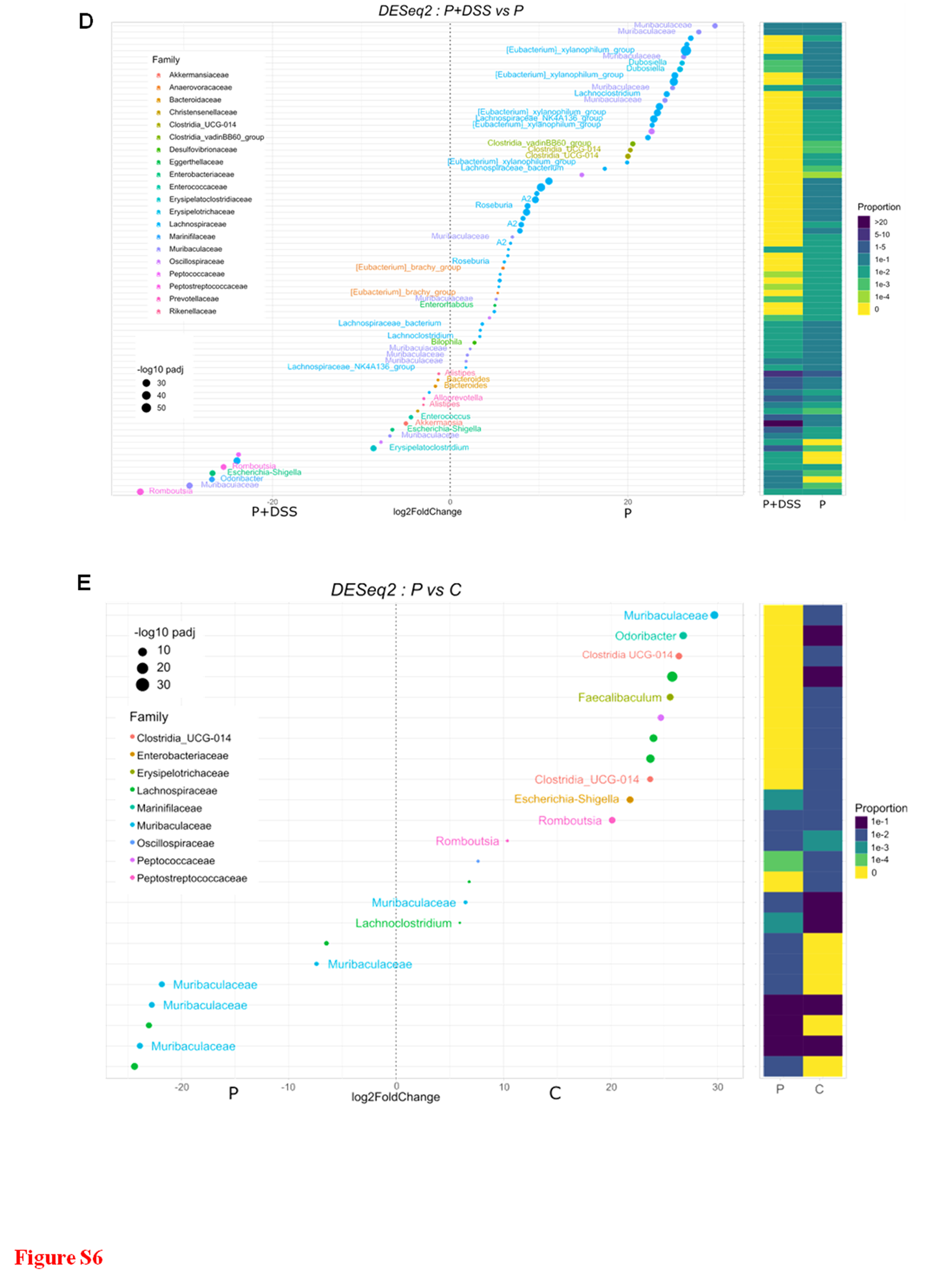

Supplement: Supplementary file 9 [file Image_9.tif]
